# Altered distribution of ATG9A and accumulation of axonal aggregates in neurons from a mouse model of AP-4 deficiency syndrome

**DOI:** 10.1371/journal.pgen.1007363

**Published:** 2018-04-26

**Authors:** Raffaella De Pace, Miguel Skirzewski, Markus Damme, Rafael Mattera, Jeffrey Mercurio, Arianne M. Foster, Loreto Cuitino, Michal Jarnik, Victoria Hoffmann, H. Douglas Morris, Tae-Un Han, Grazia M. S. Mancini, Andrés Buonanno, Juan S. Bonifacino

**Affiliations:** 1 Cell Biology and Neurobiology Branch, Eunice Kennedy Shriver National Institute of Child Health and Human Development, National Institutes of Health, Bethesda, Maryland, United States of America; 2 Section of Molecular Neurobiology, Eunice Kennedy Shriver National Institute of Child Health and Human Development, National Institutes of Health, Bethesda, Maryland, United States of America; 3 Institute of Biochemistry, Christian-Albrechts University of Kiel, Kiel, Germany; 4 Division of Veterinary Resources, National Institutes of Health, Bethesda, Maryland, United States of America; 5 NIH Mouse Imaging Facility/NIH Magnetic Resonance Facility, National Institute of Neurological Disorders and Stroke, National Institutes of Health, Bethesda, Maryland, United States of America; 6 Laboratory of Communication Disorders, National Institute on Deafness and Other Communication Disorders, National Institutes of Health, Bethesda, Maryland, United States of America; 7 Department of Clinical Genetics, Erasmus Medical Center, Rotterdam, The Netherlands; Children's Hospital of Philadelphia Research Institute, UNITED STATES

## Abstract

The hereditary spastic paraplegias (HSP) are a clinically and genetically heterogeneous group of disorders characterized by progressive lower limb spasticity. Mutations in subunits of the heterotetrameric (ε-β4-μ4-σ4) adaptor protein 4 (AP-4) complex cause an autosomal recessive form of complicated HSP referred to as “AP-4 deficiency syndrome”. In addition to lower limb spasticity, this syndrome features intellectual disability, microcephaly, seizures, thin corpus callosum and upper limb spasticity. The pathogenetic mechanism, however, remains poorly understood. Here we report the characterization of a knockout (KO) mouse for the *AP4E1* gene encoding the ε subunit of AP-4. We find that AP-4 ε KO mice exhibit a range of neurological phenotypes, including hindlimb clasping, decreased motor coordination and weak grip strength. In addition, AP-4 ε KO mice display a thin corpus callosum and axonal swellings in various areas of the brain and spinal cord. Immunohistochemical analyses show that the transmembrane autophagy-related protein 9A (ATG9A) is more concentrated in the *trans*-Golgi network (TGN) and depleted from the peripheral cytoplasm both in skin fibroblasts from patients with mutations in the μ4 subunit of AP-4 and in various neuronal types in AP-4 ε KO mice. ATG9A mislocalization is associated with increased tendency to accumulate mutant huntingtin (HTT) aggregates in the axons of AP-4 ε KO neurons. These findings indicate that the AP-4 ε KO mouse is a suitable animal model for AP-4 deficiency syndrome, and that defective mobilization of ATG9A from the TGN and impaired autophagic degradation of protein aggregates might contribute to neuroaxonal dystrophy in this disorder.

## Author summary

Hereditary spastic paraplegia (HSP) is a genetic disorder characterized by progressive stiffness of the legs. To date, mutations in more than 70 genes have been shown to cause HSP. Four of these genes encode the subunits of adaptor protein 4 (AP-4), a heterotetrameric complex involved in intracellular protein trafficking. AP-4 deficiency belongs to a subset of complicated HSPs featuring intellectual disability, seizures, microcephaly and thinning of the corpus callosum in addition to leg stiffness. Here we describe the characteristics of mice lacking the expression of the ε subunit of AP-4. These mice exhibit motor deficits consistent with those seen in the human patients. In addition, they have an abnormally thin corpus callosum and defective axons in various parts of the brain and the spinal cord. Importantly, fibroblasts from AP-4 deficient patients as well as neurons from AP-4 deficient mice fail to deliver the autophagy-related protein 9A (ATG9A) from the Golgi complex to the cell periphery. This defect is associated with accumulation of abnormal protein aggregates in axons from AP-4-deficient neurons. We conclude that AP-4 mutant mice are a suitable animal model for studies of AP-4 deficiency in humans, and that altered localization of ATG9A and aggregate formation in neurons likely contribute to the development of the disease.

## Introduction

The hereditary spastic paraplegias (HSPs) are a clinically and genetically diverse group of neurological disorders characterized by progressive lower limb spasticity [1, 2, 3]. HSPs are further classified into “pure” and “complicated” depending on the absence or presence of additional clinical features [1, 2, 3]. The lower limb spasticity results from degeneration of the long axons of motor neurons in corticospinal tracts, whereas the additional features derive from dysfunction of other neurons or glial cells [1, 2, 3]. To date, more than 70 genetic loci (designated SPG, for “spastic paraplegia”) have been linked to HSP. Four of these loci encode the subunits of the adaptor protein 4 (AP-4) complex [4, 5], namely SPG47 (OMIM #614066) (*AP4B1*/β4), SPG50 (OMIM #612936) (*AP4M1*/μ4), SPG51 (OMIM #613744) (*AP4E1*/ε) and SPG52 (OMIM #614067) (*AP4S1*/σ4) [6, 7, 8, 9, 10, 11, 12]. Collectively, these four loci define a subset of HSPs referred to as “AP-4 deficiency syndrome”, which is inherited in an autosomal recessive manner and has characteristics of complicated HSP because of the presence of intellectual disability, microcephaly, seizures, growth retardation, thin corpus callosum and upper limb spasticity in addition to lower limb spasticity [6, 7, 8, 9, 10, 11, 12].

AP-4 belongs to a family of heterotetrameric adaptor protein (AP) complexes involved in protein sorting in the endomembrane system of eukaryotic cells [13, 14]. In mammals, AP-4 is ubiquitously expressed in various cells and tissues [4, 5], including different areas of the brain [15] (http://www.brain-map.org/). At the subcellular level, AP-4 localizes to the *trans*-Golgi network (TGN) [4, 5] through association with Arf-family small GTPases [16]. At this location, AP-4 has been proposed to sort cargos, such as members of the amyloid precursor protein (APP) family [17], amino-3-hydroxy-5-methyl-4-isoxazolepropionic acid (AMPA)-type glutamate receptors (AMPAR) [18], low density lipoprotein receptor (LDLR) [18] and δ2 glutamate receptor (δ2R) [15, 18], into transport carriers destined for post-Golgi compartments of the endomembrane system. Homozygous disruption of the gene encoding the β4 subunit of AP-4 in mouse resulted in animals with apparently milder symptoms than those of human SPG47 patients, with the only overt abnormality being poor performance in the rotarod test [18]. Nevertheless, immunohistochemical staining of brain sections and cultured neurons from the β4 knockout (KO) mice revealed partial redistribution of the somatodendritic AMPAR, LDLR and δ2R to the axon of Purkinje and hippocampal neurons [18]. On the basis of these observations, it was proposed that AP-4 functions in the polarized sorting of a subset of cargos to the somatodendritic domain [18]. Intriguingly, the missorted AMPAR did not localize to the surface of the axon but to axonal autophagosomes labeled for the autophagic protein microtubule-associated proteins 1A/1B light chain 3B (LC3B) [18]. These autophagosomes were often found within large bulges or spheroids near axon terminals [18]. AMPAR accumulation within these structures could be the manifestation of a mechanism to dispose of missorted somatodendritic proteins by autophagy. However, it could also reflect a more direct role of AP-4 in autophagy. In this regard, we recently showed that KO or knockdown (KD) of AP-4 subunits in non-neuronal cells impaired export of the transmembrane autophagy-related protein 9A (ATG9A) from the TGN towards peripheral compartments such as endosomes and pre-autophagosomal structures (PAS) [19]. This finding raised the possibility that at least some of the neuronal phenotypes of AP-4-deficient mice result from altered autophagy.

To gain further insight into the pathogenesis of AP-4 deficiency syndrome, we investigated the effects of disrupting the gene encoding another subunit of AP-4, *AP4E1*/ε, in the mouse. We found that homozygous AP-4 ε KO mice not only had impaired performance on the rotarod, but also exhibited other motor and behavioral abnormalities including hindlimb clasping, reduced grip strength, increased ambulation and enhanced acoustic startle response. Furthermore, magnetic resonance imaging (MRI) and histological studies revealed the presence of an abnormally thin corpus callosum, a hallmark of AP-4 deficiency syndrome in humans [6, 7, 8, 9, 10, 11, 12]. Immunohistochemical analyses also showed the presence of axonal spheroids in various regions of the brain. Importantly, we found that ATG9A was more concentrated at the TGN and depleted from the peripheral cytoplasm in both skin fibroblasts from patients with mutations in AP-4 μ4 and neurons from AP-4 ε KO mice, as compared to their normal counterparts. In neurons, this redistribution of ATG9A was associated with an increased tendency to accumulate an aggregation-prone huntingtin mutant in the axon. These findings thus establish AP-4 ε KO mice as a suitable animal model for human AP-4 deficiency syndrome, and indicate that mislocalization of ATG9A and impaired degradation of intracellular aggregates might contribute to the pathogenesis of this disorder.

## Results

### Molecular characterization of AP-4 ε KO mice

**Fig 1:**
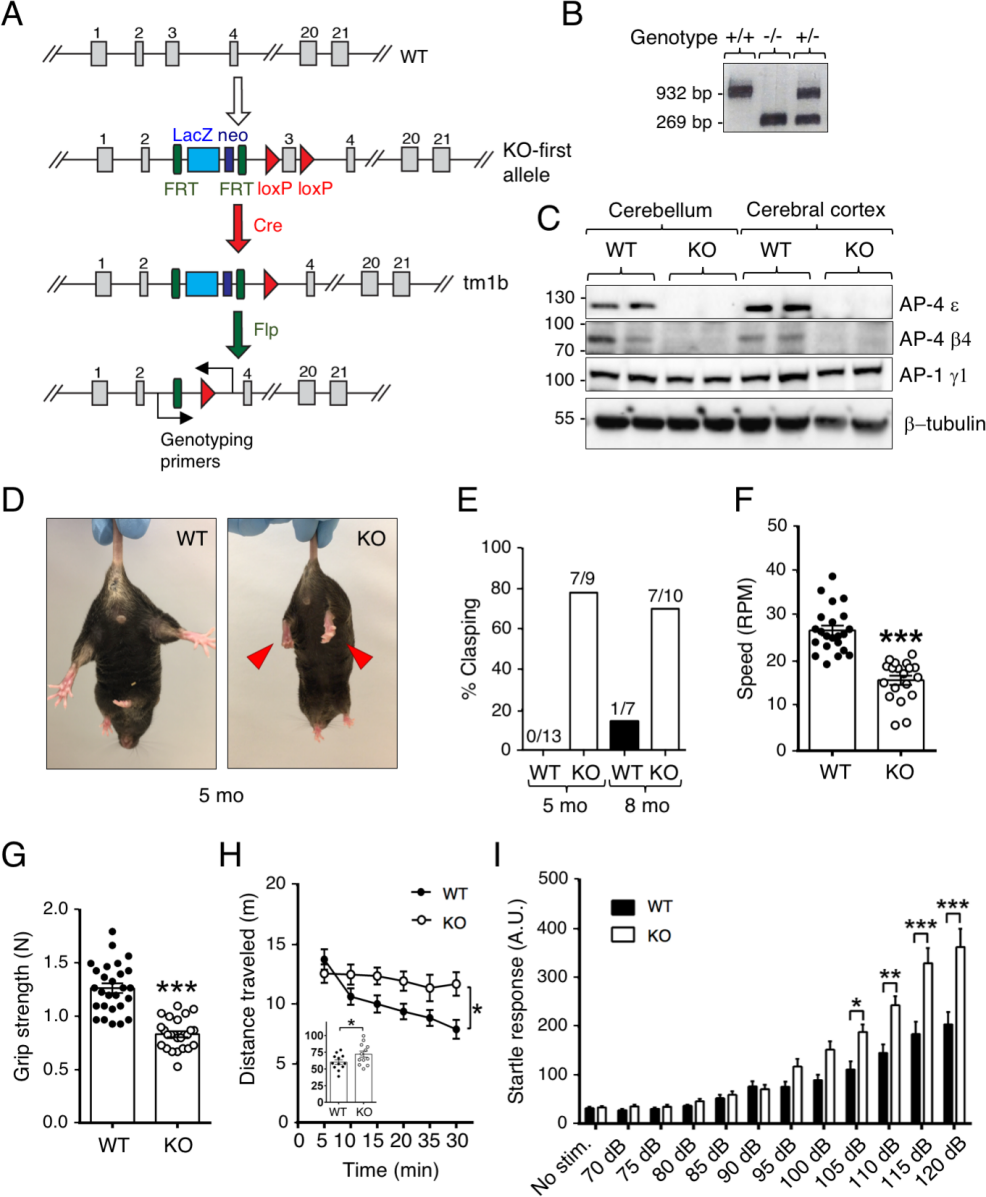
Molecular and behavioral characteristics of AP-4 ε KO mice. (A) Schematic representation of the strategy for generation of the AP-4 ε (*AP4E1*) KO mouse. Exons are numbered. Exon 3 was deleted by Cre-mediated recombination, resulting in a null allele caused by a frameshift and premature termination of the ORF. The neomycin cassette used for selection, as well as Lac Z reporter sequences, were removed by Flp-mediated recombination. The positions of the PCR primers used for genotyping are indicated. (B) Genotyping of +/+ (WT), -/- (KO) and +/- (heterozygous) mice was done using PCR primers complementary to sequences flanking exon 3. The WT and KO alleles generated 932 and 269 bp fragments, respectively. (C) SDS-PAGE and immunoblot analysis of AP-4 ε and β4 subunits, AP-1 γ1 subunit, and β-tubulin (control) in homogenates of cerebellum and cerebral cortex from two WT and two KO mice. The positions of molecular mass markers (in kDa) are indicated on the left. (D) Representative picture of the clasping response of 5-month-old WT and KO mice held by the tail. Clasping of hind paws in the KO mouse is indicated by red arrowheads. (E) Percentage of WT and KO mice that show clasping at 5 and 8 months (mo) of age. Numbers on top of each bar indicate number of clasping/total mice in each group (5 mo WT: 5 male, 8 female, KO: 4 males, 5 females; 8 mo WT: 4 male, 3 female, KO: 7 male, 3 female). (F) Performance of 3-month-old mice on the rotarod. Numbers of mice used were: 22 WT (12 male, 10 female) and 19 KO (10 male, 9 female). Individual points show the RPM at which each mouse fell from the rod. Bars indicate the mean ± SEM for each group. (G) Grip strength of 3-month-old mice. Numbers of mice used were: 27 WT (13 male, 14 female) and 25 KO (13 male, 12 female). Individual points show the grip strength in Newton (N) and bars indicate the mean ± SEM for each group. (H) Novelty-induced locomotor activity of 3-month-old mice in the open field test. Numbers of mice used were: 12 WT (6 male, 6 female) and 12 KO (6 male, 6 female). Values are the mean ± SEM of the distance traveled every 5 min. The inset describes the cumulative total distance traveled in meters (m) during a total of 30 min. (I) Acoustic startle response of 3-month-old mice subjected to increased noise levels in decibels (dB). Numbers of mice used were: 22 WT (11 male, 11 female) and 22 KO (10 male, 12 female). Values are the mean ± SEM of the startle response in arbitrary units (A.U.). *P<0.05, **P<0.005, ***P<0.0005.

C57BL/6 mice with a deletion of exon 3 of the *AP4E1* gene (tmb1, Fig 1A), encoding the AP-4 ε subunit, were obtained from the UC Davis KOMP repository (https://www.komp.org/). These mice were bred to a site-specific Flp deleter strain to remove the LacZ and neomycin reporter cassette in all tissues (Fig 1A). The genotype of +/+ (WT), -/- (KO) and +/- (heterozygous) mice was determined by polymerase chain reaction (PCR) using primers for sequences flanking exon 3 (Fig 1A and 1B). Deletion of exon 3 is predicted to cause a frameshift after amino acid 114 and the addition of 41 extraneous amino acids at the C-terminus of the truncated protein. In agreement with this prediction, immunoblot analysis of samples of cerebellum and cerebral cortex showed a complete absence of the ~125-kDa AP-4 ε protein in AP-4 ε KO mice (Fig 1C). The levels of the ~83-kDa AP-4 β4 subunit were also drastically reduced in the AP-4 ε KO samples (Fig 1C), consistent with the degradation of this subunit when AP-4 ε is missing. In contrast, the levels of the γ1 subunit of the AP-1 complex were unchanged in the AP-4 ε KO mice (Fig 1C). These results were consistent with KO of the ε subunit causing a specific depletion of the entire AP-4 complex.

### Motor and behavioral deficits of AP-4 ε KO mice

Mating of heterozygous AP-4 ε KO mice produced offspring at expected Mendelian ratios. Homozygous KO mice did not show any overt phenotypes, had a normal life span and were fertile. Gross anatomy, weight of major organs and hematological parameters were also not significantly altered. However, we noticed that AP-4 ε KO mice exhibited an abnormal phenotype of hindlimb clasping when held by the tail (Fig 1D and 1E). This phenotype is a common manifestation of various brain and spinal cord pathologies [20], which prompted us to investigate in further detail the motor and behavioral functions of these mice.

Motor coordination and balance were assessed using the rotarod test, which measures the speed at which mice fall from an accelerating rotating cylinder (from 4 to 40 revolutions per minute (RPM) over a 5-min period). We observed that AP-4 ε KO mice fell at lower speeds than WT mice (KO: 15.7 ± 1.0 RPM, n = 19; WT: 26.9 ± 1.0 RPM, n = 22; t(39) = 7.335, P<0.0005) (Fig 1F). We also found that AP-4 ε KO mice had reduced grip strength on a 45°-angled grid relative to WT mice (KO: 0.84 ± 0.03 N, n = 25; WT: 1.27 ± 0.05 N, n = 27; t(50) = 8.004, P<0.0005) (Fig 1G). Additional tests showed that AP-4 ε KO mice did not habituate to novel environments in the open field test as compared to WT mice (F(1,22) = 4.43, P = 0.047), as manifested by the absence of a gradual reduction in distance traveled during the 30 min test, and the longer distances traveled by the AP-4 ε KO mice relative to the WT mice (KO: 72.1 ± 4.6 m, n = 12; WT: 60.2 ± 3.3 m, n = 12; t(22) = 2.105, P = 0.047) (Fig 1H). The increased locomotor activity observed in the AP-4 ε KO mice is likely to be unrelated to altered anxiety or risk-taking behaviors, as WT and AP-4 ε KO mice spent similar percentages of times exploring the open arms of the elevated plus maze (KO: 8.5 ± 1.5%, n = 8; WT: 9.3 ± 1.7%, n = 9; P>0.05, *i*.*e*., not significant) (S1A Fig). AP-4 ε KO mice also showed enhanced startle motorsensory response to loud acoustic stimuli at ≥105 dB (KO: n = 22; WT n = 22; F(1,504) = 61.32, P<0.0005) (Fig 1I). In contrast to the above phenotypes, we could not detect differences between WT and AP-4 ε KO mice in tasks relevant to working, learning and spatial memory in the Barnes (S1B Fig) and T-maze tests (S1C Fig). Taken together, our findings indicated the AP-4 ε KO mice exhibit a range of motor and behavioral abnormalities that are consistent with at least some of the clinical features of AP-4-deficient patients [6, 7, 8, 9, 10, 11, 12].

### Thin corpus callosum and cerebellar spheroids in AP-4 ε KO mice

**Fig 2:**
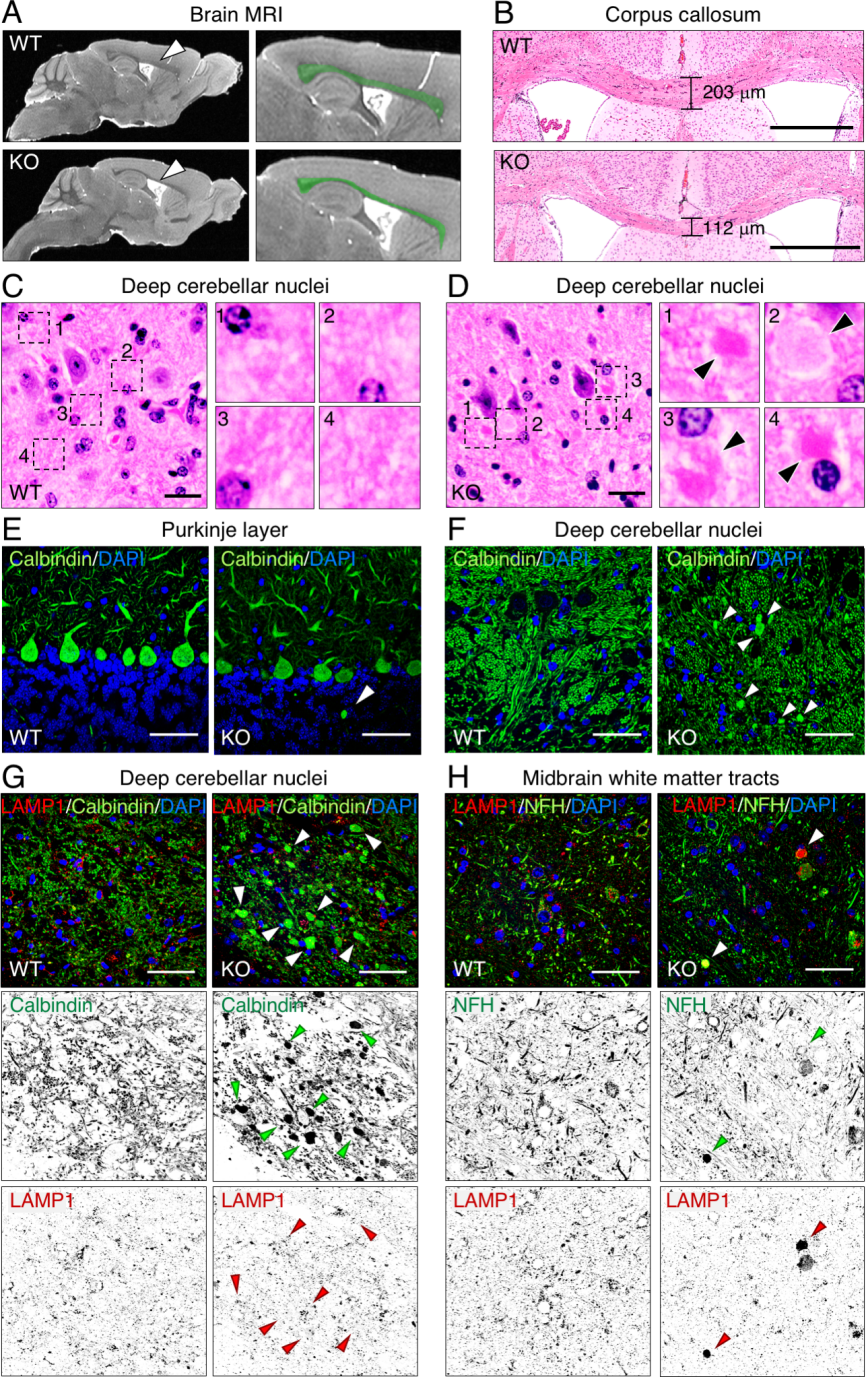
Thin corpus callosum and axonal spheroids in the brain of AP-4 ε KO mice. (A) MRI shows thinner corpus callosum (arrowheads and green highlights) in the brain of a 9-month-old KO relative to an age-matched WT mouse. Results are representative of three mice per group. (B) Coronal brain sections stained with H&E show ~55% thinner corpus callosum in a 9-month-old KO mouse relative to an aged-matched WT mouse. Bars: 500 μm. The thickness of the corpus callosum in comparable sections is indicated. Results are representative of two mice per group. (C,D) H&E staining of coronal sections of brains from 9-month-old mice show the appearance of both weakly and strongly stained spheroids (arrowheads) in deep cerebellar nuclei (DCN) of KO but not WT mice. The numbered panels are four-fold magnifications of the boxed areas. Bars: 20 μm. (E,F) Immunohistochemical staining for calbindin (green) in sections from the cerebellum shows rare axonal spheroids in the proximal axons of Purkinje neurons (E) and numerous spheroids in distal axons of Purkinje neurons in DCN (F) of KO mice but not WT mice. Spheroids are indicated by arrowheads. Bars: 50 μm. (G) Immunohistochemical co-staining for calbindin (green) and LAMP1 (red) of sections from the cerebellum show that Purkinje cells spheroids in DCN from KO mice are negative for LAMP1 (arrowheads). Bars: 50 μm. (H) Immunohistochemical co-staining for the neurofilament protein NFH (green) and LAMP1 (red) of sections from midbrain white matter tracts show spheroids containing both proteins (arrowheads) in KO mice. Bars: 50 μm. In G and H, single-channel images are shown in inverted grayscale. In E-H, DAPI (blue) was used to stain the nuclei.

Brain MRI showed that the corpus callosum was thinner in AP-4 ε KO mice than in WT mice (Fig 2A, arrows and green highlights). Other brain structures in the AP-4 ε KO mice appeared normal at this level of resolution (Fig 2A). Hematoxylin and eosin (H&E) staining of brain sections also showed reduced thickness of the corpus callosum in KO mice relative to WT mice (Fig 2B). In contrast, the thickness of the cerebral cortex was unaltered in the KO mice (S2 Fig). H&E staining additionally showed the presence of both strongly and weakly stained spheroid-like bodies of up to 20 μm in diameter in the neuropil of deep cerebellar nuclei (DCN) in AP-4 ε KO but not WT mice (Fig 2C and 2D). H&E staining of sections from other brain regions or other organs did not reveal any appreciable differences between AP-4 ε KO and WT mice. The fact that the AP-4 ε KO mice have a thin corpus callosum like AP-4 deficient patients further supports the correspondence of this mouse model to the human disease [6, 7, 8, 9, 10, 11, 12].

### Widespread axonal swellings in the brain of AP-4 ε KO mice

To search for more specific abnormalities in the brain of AP-4 ε KO relative to WT mice, we performed immunostaining of brain sections. Staining with an antibody to the Purkinje neuron marker calbindin showed a normal number and appearance of Purkinje neurons in AP-4 ε KO relative to WT mice (Fig 2E, S3 Fig). However, occasional calbindin-positive spheroids were observed in the proximal axonal field of the Purkinje neurons from AP-4 ε KO but not WT mice (Fig 2E, arrowhead). Calbindin-positive spheroids were much more numerous in the distal axons of Purkinje neurons that project into the DCN of the AP-4 ε KO mice (Fig 2F, arrowheads). These spheroids likely correspond to those described above for H&E staining (Fig 2D). DCN spheroids were not enriched in lysosomes containing the lysosome-associated membrane protein 1 (LAMP1) (Fig 2G, arrowheads). White matter tracts in the midbrain of AP-4 ε KO mice also showed spheroids, but these did co-stain for LAMP1 and the axonal non-phosphorylated neurofilament H (NFH) protein (Fig 2H). Large axonal swellings (up to 15 μm in diameter) positive for LAMP1 were additionally found in sections of the hippocampus and spinal cord from the AP-4 ε KO mice (S4A Fig). Numerous swellings along the axon could also be observed by phase-contrast imaging of cultured hippocampal neurons from the AP-4 ε KO mice (S4B and S4C Fig). Transmission electron microscopy of plastic-embedded sections of DCN showed the normal appearance of myelinated axons of Purkinje neurons from WT mice and abnormally enlarged myelinated swellings containing a proliferation of tightly packed membrane cisternae and organelles with the appearance of mitochondria and autophagosomes in AP-4 ε KO mice (S4D Fig). These analyses thus revealed that AP-4 ε KO causes the development of axonal swellings in different neuronal types and different regions of the central nervous system (CNS). Some of these swellings contain lysosomes while others do not, suggesting that they are heterogeneous in nature. Both the thin corpus callosum described above (Fig 2A and 2B) and the widespread neuroaxonal dystrophy evidenced by the spheroids (Fig 2C–2H, S4 Fig) are likely contributors to the motor and behavioral deficits of AP-4 ε KO mice.

### Glutamate receptor distribution in Purkinje neurons from AP-4 ε KO mice

**Fig 3:**
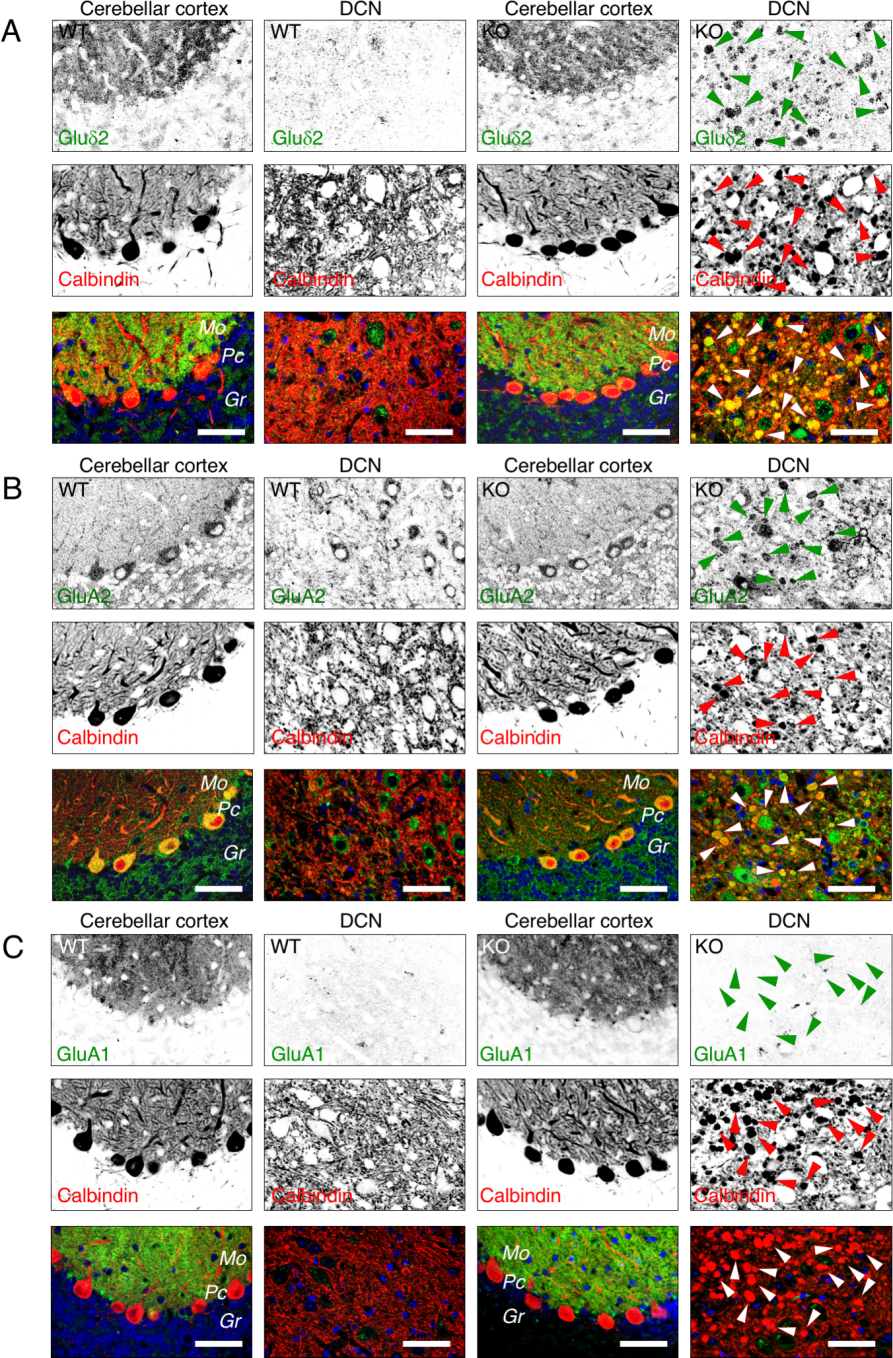
Distribution of glutamate receptors in Purkinje neurons of the cerebellum from WT and AP-4 ε KO mice. (A-C) Sections of the cerebellum from WT and AP-4 ε KO mice were co-immunostained with antibodies to calbindin (red) and δ2R (AB_2571601 from Frontier Institute Co., ltd) (A), GluA2 (SAB4501295 from Sigma) (B), or GluA1 (AB1504 from Millipore) (C). Staining for δ2R was done on sections treated with pepsin and staining for GluA1 on sections treated at 85°C with sodium-citrate buffer, in both cases for antigen retrieval. Similar results were obtained using samples that were not treated with high temperature sodium-citrate buffer (S5C and S5D Fig) and using a different antibody to GluA1 (antibody ab31232 from Abcam) (S5A and S5B Fig). Mo: molecular layer; Gr: granular layer, Pc: Purkinje cell layer, DCN: deep cerebellar nucleus. DAPI (blue) was used to stain the nuclei. Bars: 50 μm. Examples of spheroids are indicated by arrowheads. Single-channel images are shown in inverted grayscale. Notice the presence of δ2R and GluA2, and the absence of GluA1, in calbindin-positive spheroids in the DCN of AP-4 ε KO.

To investigate if the neurological phenotypes of AP-4 ε KO mice could be due to loss of the somatodendritic polarity of glutamate receptors, as previously reported for AP-4 β4 KO mice [18], we examined the localization of the endogenous δ2R (also known as GluD2 or GRID2), and the AMPAR subunits GluA2 (also known as GluR2 or GRIA2), and GluA1 (also known as GluR1 or GRIA1) in Purkinje neurons of the cerebellum (Fig 3A–3C; S5 Fig). These neurons are ideally suited for visualization of dendrite-axon polarity *in situ* because all the dendrites are oriented towards the molecular layer (Mo) and all the axons towards the granular layer (Gr) of the cerebellar cortex (Fig 3A–3C). As described above, immunohistochemistry of cerebellar sections showed numerous calbindin-positive spheroids indicative of distal swellings of Purkinje axons in DCN of AP-4 ε KO mice (Fig 3A–3C, arrowheads). Immunostaining for the δ2R showed that in both WT and AP-4 ε KO mice the receptor was predominantly concentrated in the soma and dendritic field of the Purkinje neurons (Fig 3A). However, δ2R staining could also be observed in calbindin-positive DCN spheroids from AP-4 ε KO but not WT mice (Fig 3A, arrowheads). Immunostaining for GluA2 was too weak to determine the polarity of this AMPAR subunit in Purkinje neurons, although we could observe the presence of this subunit in calbindin-positive DCN spheroids from AP-4 ε KO but not WT mice (Fig 3B, arrowheads). Similar analyses of GluA1, with (Fig 3C) or without antigen retrieval (S5 Fig) and using two different antibodies (S5 Fig), revealed that this AMPAR subunit was polarized to the somatodendritic domain of Purkinje neurons and completely absent from calbindin-positive DCN spheroids in both WT and AP-4 ε KO mice. The only GluA1 staining visible in DCN corresponded to the soma and dendrites of some neurons in this region of the cerebellum (Fig 3C; S5 Fig). Analyses of the localization of endogenous GluA1 and transgenic GluA1 tagged with green fluorescent protein (GFP) in day-in-vitro 10 (DIV10) hippocampal neurons in primary culture showed that this AMPAR subunit was also largely restricted to the somatodendritic domain and not detectably present in axonal swellings of both WT and AP-4 ε KO neurons (S6 Fig). Our experiments thus showed that axonal spheroids in AP-4 ε KO mice contain mislocalized δ2R and GluA2, but not GluA1.

### Altered distribution of ATG9A in fibroblasts from AP-4 μ4 mutant patients and neurons from AP-4 ε KO mice

**Fig 4:**
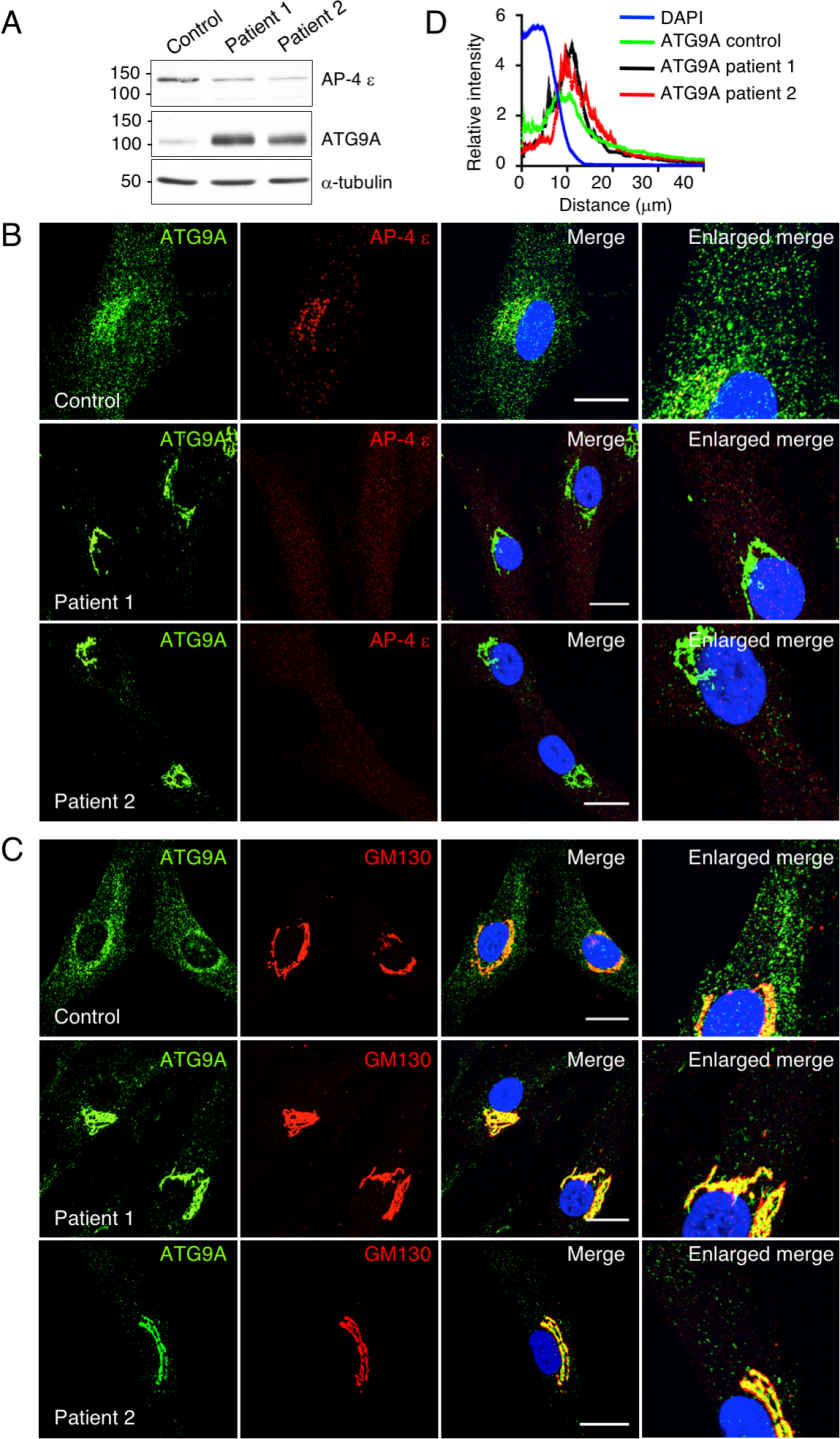
Accumulation of ATG9A at the TGN of AP-4 μ4 mutant patient fibroblasts. (A) Skin fibroblasts from one control individual and two patients homozygous for mutations in the *AP4M1* gene encoding AP-4 μ4 [7] were analyzed by SDS-PAGE and immunoblotting for the ε subunit of AP-4, ATG9A, and α-tubulin (loading control). The positions of molecular mass markers (in kDa) are indicated on the left. (B,C) Co-immunostaining for endogenous ATG9A (green) and AP-4 ε (red) (B) or GM130 (red) (C) of the fibroblasts mentioned in A. Bars in B and C: 20 μm. DAPI (blue) was used to stain the nuclei. (D) Quantification of the distribution of ATG9A relative to the nucleus in fibroblasts from control (n = 9), patient 1 (n = 7) and patient 2 (n = 7) using ImageJ with the Radial Profile plugin. Values are the mean ± SEM of fluorescence intensity relative to the total cells intensity in each group. Notice the concentration of ATG9A at the TGN and its depletion from the peripheral cytoplasm in the patients’ fibroblasts.

We next considered the possibility that AP-4 deficiency led to alterations in not only glutamate receptor localization but also in the autophagy machinery itself. In this regard, we recently reported a defect in export of the autophagy protein ATG9A from the TGN in non-neuronal human cell lines and mouse embryonic fibroblasts (MEF) deficient in AP-4 [19]. To assess the relevance of these findings to patients with AP-4 deficiency syndrome, we examined the distribution of ATG9A in skin fibroblasts from one control individual and two patients with homozygous, inactivating mutations in the *AP4M1* gene encoding the μ4 subunit of AP-4 [7] (Fig 4). Because we do not have a suitable antibody to AP-4 μ4, we could not test for the expression of this subunit by immunoblotting. However, immunoblotting for AP-4 ε showed reduced levels of this subunit in the patients’ fibroblasts (Fig 4A), consistent with the previously reported degradation of unassembled subunits of AP complexes when one subunit is mutated or missing [21]. Moreover, no immunofluorescent staining for AP-4 ε at the TGN was detected in the patients’ fibroblasts (Fig 4B). Immunoblot analysis also showed a marked increase in ATG9A levels (Fig 4A). Immunostaining for ATG9A in comparison to AP-4 ε or the Golgi matrix protein 130 (GM130) showed that whereas control fibroblasts exhibited ATG9A staining at the TGN as well as puncta distributed through the cytoplasm, μ4 mutant fibroblasts displayed brighter staining at the TGN and depletion from the peripheral cytoplasm (Fig 4B–4D). Larger fields of cells illustrating the generality of this phenotype are shown in S7 Fig.

**Fig 5:**
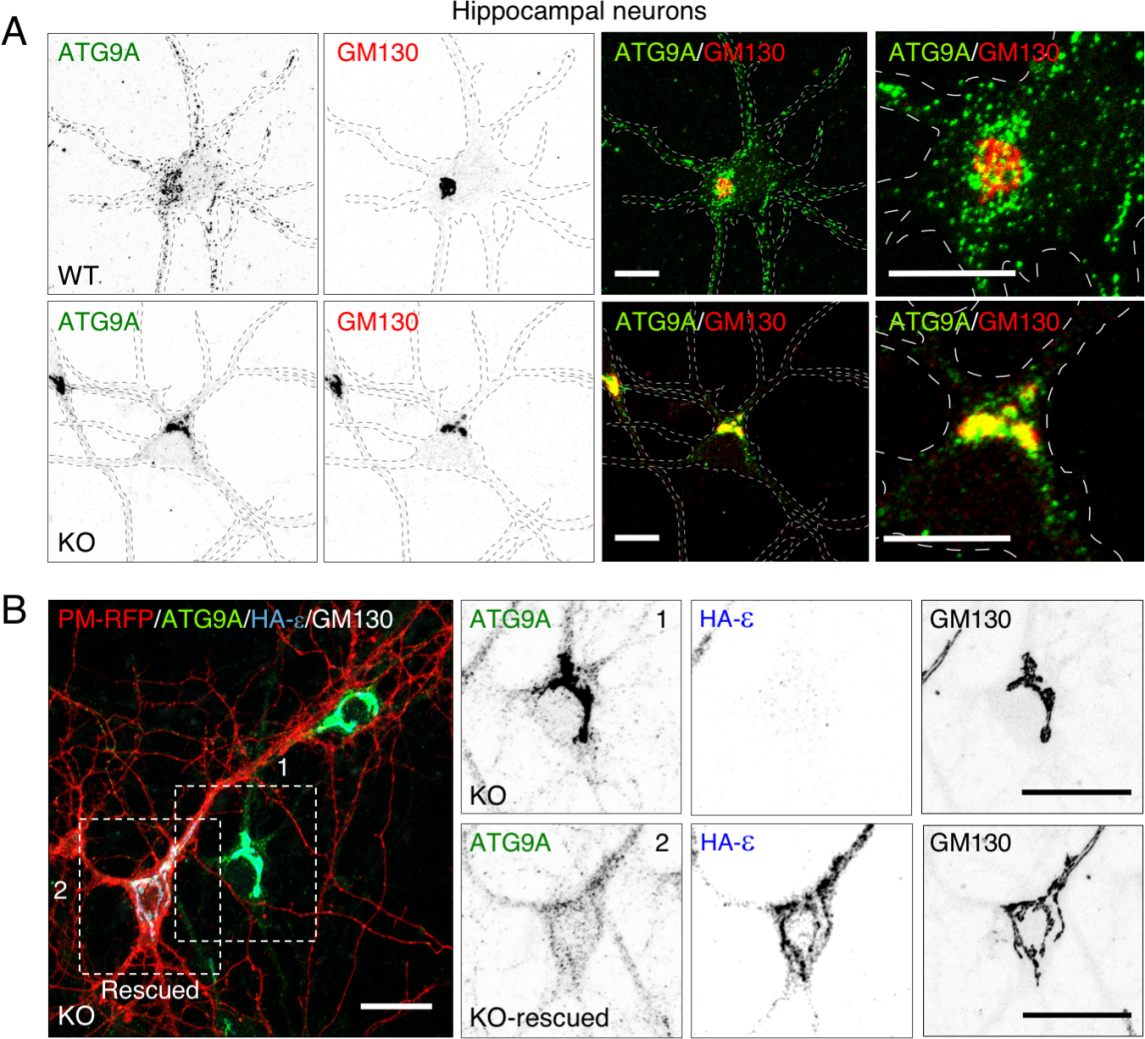
Accumulation of ATG9A at the TGN in neurons from AP-4 ε KO mice. (A) Co-immunostaining of hippocampal neurons from WT and KO mice for ATG9A (green) and GM130 (red). Single-channel images are shown in inverted grayscale. Outlines of the neurons are indicated by dashed lines. Images on the rightmost column are 3-fold-magnified views of the neuronal soma from the merged images. Bars: 10 μm. (B) Rescue of the normal distribution of endogenous ATG9A by transfection of plasmids encoding HA-tagged ε and PM-RFP into hippocampal neurons from KO mice. Neurons were stained for endogenous ATG9A (green) and GM130 (white), and for the HA epitope (blue). The six panels on the right are single-channel, inverted grayscale, magnified views of the boxed areas 1 and 2 on the left. Bar: 20 μm. Notice the concentration of ATG9A at the TGN and concomitant depletion from the periphery in the AP-4 ε KO neurons, and the reversal of this phenotype in the HA-ε-rescued neurons.

In DIV9 WT mouse hippocampal neurons in primary culture, ATG9A also localized to both the TGN in the cell body and numerous foci scattered throughout the cytoplasm, including dendrites and axon (Fig 5A). In contrast, in AP-4 ε KO neurons ATG9A was exclusively found at the TGN and depleted from peripheral locations (Fig 5A). The non-polarized distribution of the lysosomal marker LAMP1-GFP to dendrites and axon [22] and the axonal polarity of the synaptic vesicle marker RAB3A-GFP [23] were not altered in AP-4 ε KO relative to WT neurons (S8A Fig), indicating that AP-4 depletion did not prevent transport of organelles into the axon and dendrites or cause a generalized defect in polarized sorting. Rescue of KO neurons with a plasmid encoding HA-tagged ε (HA-ε) decreased the concentration of ATG9A at the TGN and restored its peripheral distribution (Fig 5B).

**Fig 6:**
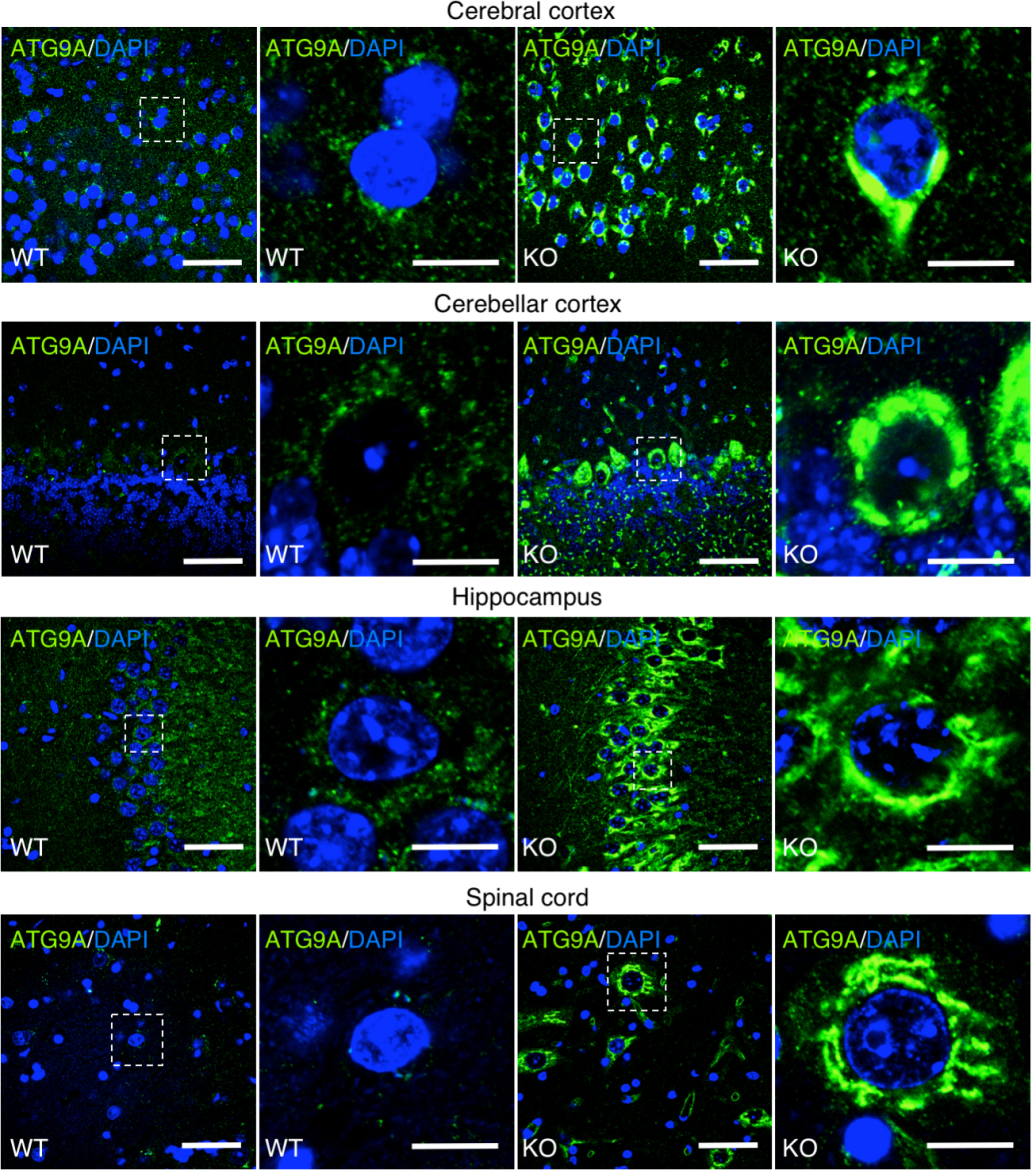
Accumulation of ATG9A at the TGN in different brain regions from AP-4 ε KO mice. Immunostaining of sections from the cerebral cortex, cerebellar cortex, hippocampus, and spinal cord of WT and AP-4 ε KO mice using an antibody to endogenous ATG9A (green). Nuclei were stained with DAPI (blue). Images on the second and fourth columns are magnifications of the boxed areas on the first and third columns, respectively. Bars: 50 μm (zoom-out view) and 10 μm (magnification). Notice the brighter ATG9A staining at the TGN in neurons from AP-4 ε KO mice.

Immunostaining of sections from the cerebral cortex, cerebellar cortex, hippocampus and spinal cord (Fig 6) also showed dispersed cytoplasmic staining for ATG9A in neurons from WT mice and brighter staining at the TGN in neurons from AP-4 ε KO mice. The difference in staining of these sections was so striking that it was difficult to observe ATG9A staining in WT samples, but it was easily seen in the KO samples (Fig 6). From these experiments, we concluded that, as in non-neuronal cells (Fig 4B–4D) [19], AP-4 is required for export of ATG9A from the TGN in neurons.

### Increased levels of ATG9A but not LC3B in different regions of the brain from AP-4 ε KO mice

**Fig 7:**
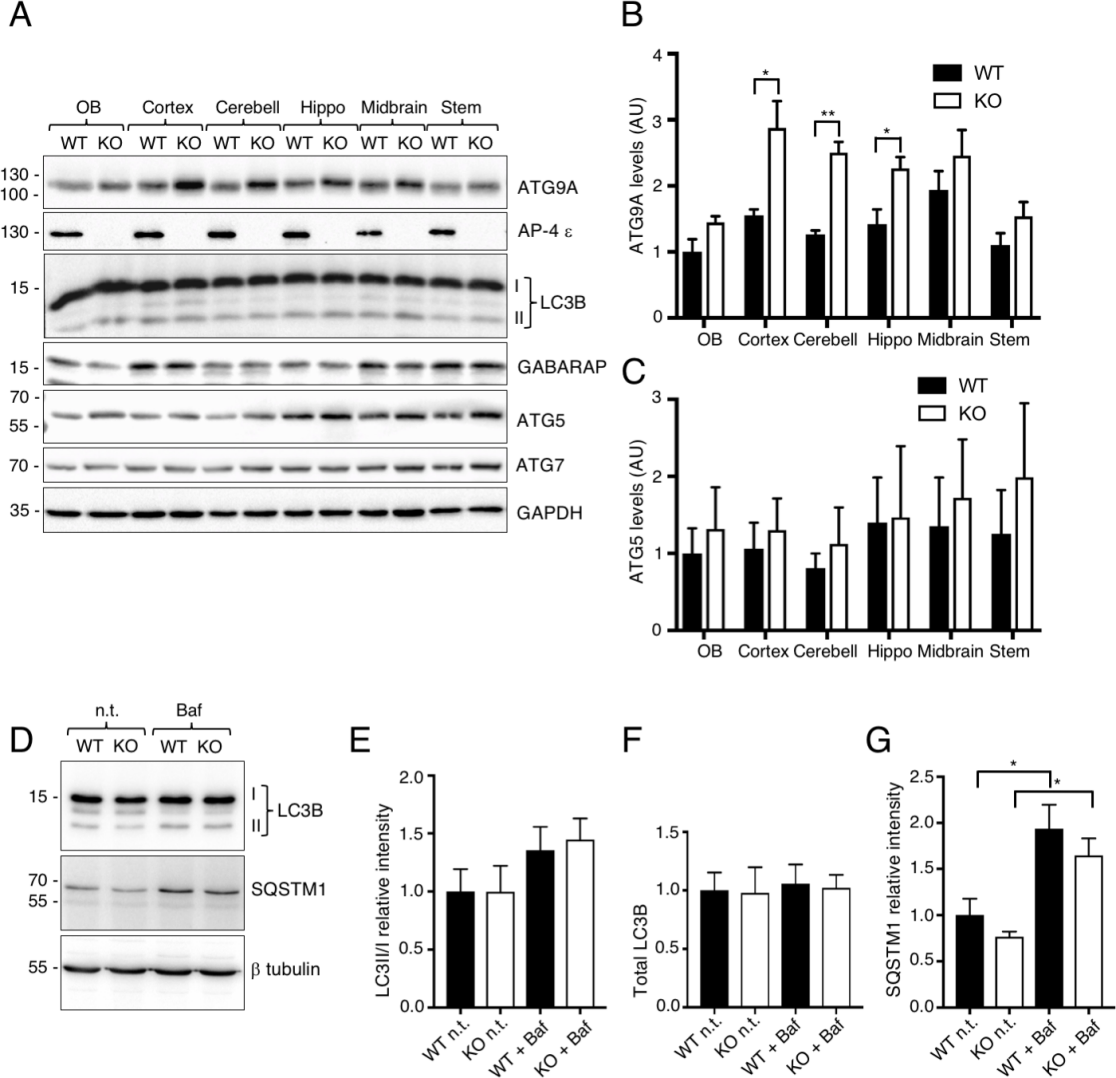
Increased levels of ATG9A and normal levels of other autophagy proteins in brain and neurons from AP-4 ε KO mice. (A) Homogenates of the olfactory bulb (OB), cerebral cortex, cerebellum (Cerebell), hippocampus (Hippo), midbrain and brain stem from WT and AP-4 ε KO mice were analyzed by SDS-PAGE and immunoblotting with antibodies to the proteins indicated on the right. The positions of the I and II forms of LC3B are indicated. The positions of molecular mass markers (in kDa) are indicated on the left. (B) Quantification of ATG9A levels from experiments as in A. Values are the mean ± SEM from four determinations. *P<0.05, **P<0.005. Notice the increased levels of ATG9A in some brain regions of AP-4 ε KO *vs*. WT mice. (C) Quantification of ATG5 levels from experiments as in A. Values are the mean ± SEM from four determinations. Notice that ATG5 levels are not significantly changed in AP-4 ε KO *vs*. WT mice. (D) Cortical neurons in primary culture from WT and AP-4 ε KO mice were incubated in the absence (n.t.) or presence of 100 nM bafilomycin A1 (Baf) for 5 h. Total proteins extracts were analyzed by SDS-PAGE and immunoblotting for LC3B, SQSTM1, and β-tubulin. The positions of the I and II forms of LC3B are indicated. The positions of molecular mass markers (in kDa) are indicated on the left. (E-G) Quantification of LC3B-II to LC3B-I ratio (E), total LC3B (F) and total SQSTM1 (G) relative to β-tubulin from experiments such as that in D. Values are the mean ± SD from three determinations. *P<0.05. Notice the significant increase of SQSTM1 after incubation with bafilomycin in both WT and AP-4 ε KO cortical neurons. Also notice that AP-4 ε KO does not affect LC3B processing and autophagic flux.

In addition to redistribution of ATG9A to the TGN, immunoblot analysis of different regions of the brain showed that ATG9A levels were significantly increased in the cerebral cortex, cerebellum and hippocampus of AP-4 ε KO relative to WT mice (Fig 7A and 7B). Both the redistribution and higher expression levels probably contributed to the much brighter staining of ATG9A at the TGN in brain and spinal cord sections (Fig 6). The levels of another autophagy protein, the autophagy-related protein 5 (ATG5), appeared slightly increased in the KO mice in the experiment shown in Fig 7A, but analysis of several experiments showed that the differences were not statistically significant (Fig 7C). The levels of other autophagy proteins such as LC3B, gamma-aminobutyric acid receptor-associated protein (GABARAP) and autophagy-related protein 7 (ATG7) were unchanged in all brain regions of AP-4 ε KO *vs*. WT mice (Fig 7A). The increase in ATG9A levels in the KO mice, as well as in the patient cells (Fig 4A), could reflect a mechanism to compensate for the inability to mobilize ATG9A from the TGN.

Upon activation of autophagy, LC3B is converted from a cytosolic LC3B-I form to a lipidated LC3B-II form that is associated with the membrane of developing autophagosomes [24]. We observed that LC3B-I was the predominant form of LC3B, and that the ratio of LC3B-II to LC3B-I was similar, in different regions of the brain (Fig 7A) as well as in cortical neurons (7D,E) from WT and AP-4 ε KO mice. This contrasted with mouse embryonic fibroblasts (MEF) from the KO animals, in which the LC3B-II to LC3B-I ratio was decreased relative to WT MEF [19]. Moreover, the total levels of LC3B and the autophagy cargo receptor sequestosome 1 (SQSTM1) (also known as p62) were not significantly different in cortical neurons from WT and AP-4 ε KO mice, and incubation with the V-ATPase inhibitor bafilomycin A1 caused similar increases in the levels of SQSTM1, an indicator of autophagic flux (Fig 7D, 7F and 7G). These observations indicated that, under basal conditions of culture, changes in the distribution of ATG9A in AP-4 ε KO neurons had little impact on the conversion of LC3B-I to LC3B-II and the degradation of LC3B-II and SQSTM1 in autolysosomes [24, 25]. This absence of obvious autophagy defects under basal conditions could be due to the compensatory increase in ATG9A levels in the AP-4 ε KO cells and tissues (Figs 4A, 7A and 7B) [19].

### Accumulation of mutant huntingtin aggregates in the axon of AP-4 ε KO neurons

**Fig 8:**
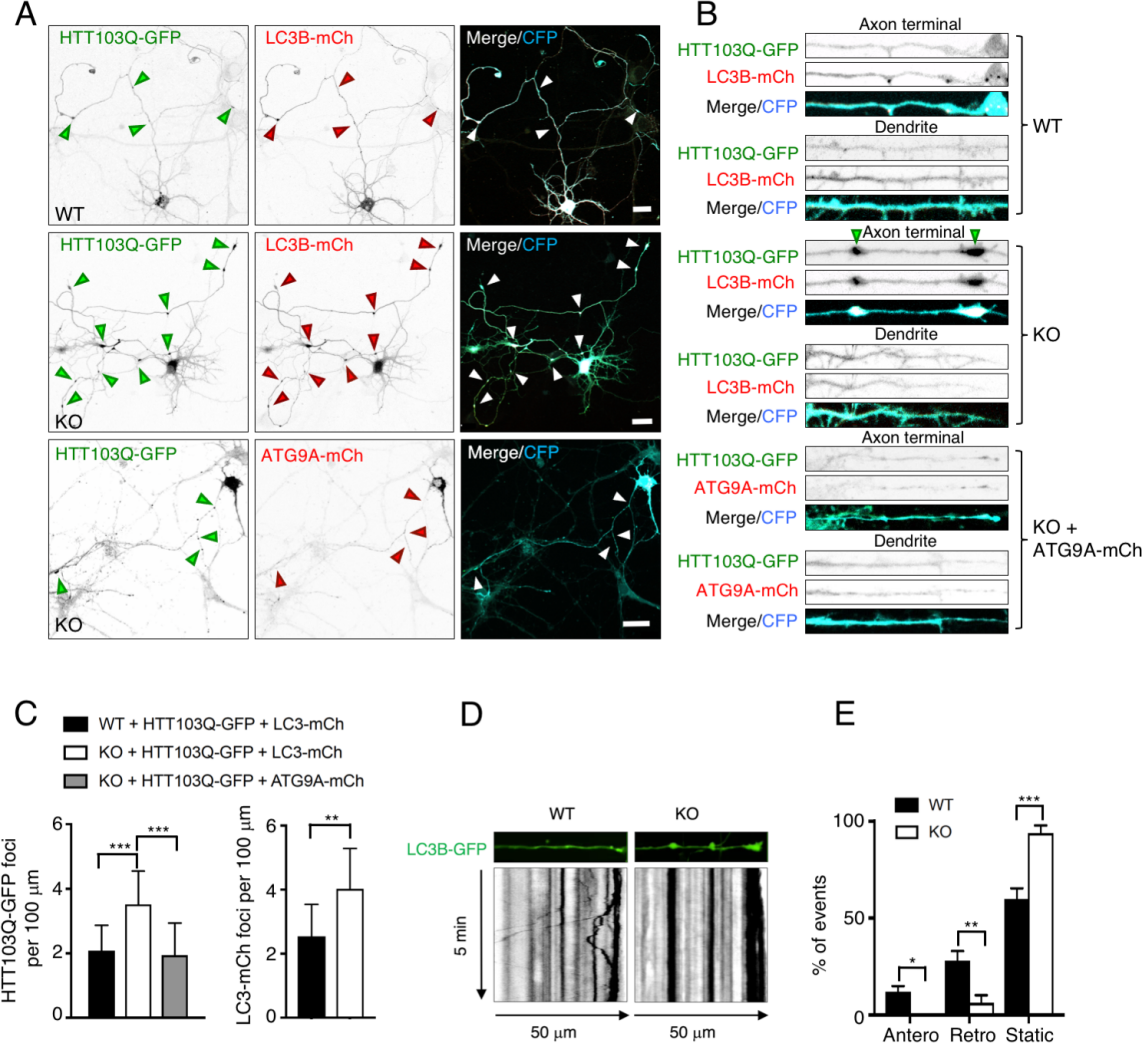
Increased accumulation of mutant huntingtin aggregates and decreased mobility of autophagosomes in the axon of AP-4 ε KO hippocampal neurons. (A) Imaging of cultured hippocampal neurons from WT and AP-4 ε KO mice co-transfected with plasmids encoding aggregation-prone HTT103Q-GFP (green) and cyan fluorescent protein (CFP) together with either LC3B-mCherry (mCh) (red) (top two rows), or ATG9A-mCherry (red) (bottom row). Single-channel images are shown in inverted grayscale. Arrowheads indicate axonal aggregates containing both GFP-and mCherry-labeled proteins. Bars: 20 μm. (B) Magnified and straightened axon terminals and dendrites from WT and AP-4 ε KO neurons shown in A. (C) Quantification of the number of HTT103Q-GFP foci with LC3B-mCherry or ATG9A-mCherry per 100 μm of axon. Values are the mean ± SD from 10 neurons. **P<0.005, ***P<0.0005. Notice the increased number of axonal HTT103Q-GFP and LC3B-mCherry aggregates in KO relative to WT neurons, and the suppression of this increase by overexpression of ATG9A-mCherry. (D) Straightened 50 μm segments of axon terminals from DIV8 WT and AP-4 ε KO hippocampal neurons expressing LC3B-GFP (top) and corresponding kymographs (bottom). Vesicles moving to the right or the left of the top panel represent anterograde or retrograde movement, respectively. Lines with negative or positive slopes in the kymographs (bottom) correspond to anterograde or retrograde movement, respectively. Note the retrogradely moving LC3B-GFP vesicles in the WT axon, and the absence of moving vesicles in the AP-4 ε KO axon. (E) Quantification of the number of anterograde, retrograde and static LC3B-GFP particles in the distal axon of WT and AP-4 ε KO neurons. The number of moving and static particles is expressed as a percentage of the total number of events in each kymograph. Values are the mean ± SEM from nine WT and five AP-4 ε KO neurons. *P<0.05, **P<0.005, ***P<0.0005.

Since the mislocalization of ATG9A in AP-4 ε KO neurons did not affect basal LC3B total levels and lipidation, we wondered if processes that depend on autophagy might be compromised under stress conditions in these cells. Neuronal autophagy is relatively insensitive to nutrient starvation [26, 27] and, indeed, we did not find significant differences in LC3B lipidation and SQSTM1 degradation in cultured hippocampal neurons from WT and KO mice subjected to amino-acid and serum withdrawal. Another process that is dependent on autophagy is the clearance of aggregates of damaged or mutant proteins, a process that is particularly critical for maintenance of axonal health [28, 25, 29]. To test for possible defects in aggregate clearance, we co-transfected WT and AP-4 ε KO hippocampal neurons with plasmids encoding LC3B-mCherry and an aggregation-prone N-terminal fragment of huntingtin (HTT) with an expanded polyglutamine tract tagged with GFP (HTT103Q-GFP) [28, 30]. These experiments revealed a significantly increased number of axonal swellings containing both HTT103Q-GFP and LC3B-mCherry in AP-4 ε KO relative to WT neurons (Fig 8A–8C). We concluded that AP-4 ε KO neurons have an impaired ability to dispose of cytoplasmic aggregates in the axon. The axonal accumulation of HTT103Q-GFP and LC3B-mCherry was not likely due to lysosomal dysfunction, since lysosomes from WT and AP-4 ε KO neurons were equally stained with the acidic compartment indicator LysoTracker and the lysosomal degradation probe DQ-BSA (S8B and S8C Fig). Overexpression of ATG9A-mCherry reduced the number of HTT103Q-GFP foci in the axon of AP-4 ε KO neurons (Fig 8A–8C), supporting the conclusion that the axonal accumulation of HTT103Q-GFP was due to a deficit in the delivery of endogenous ATG9A to the axon. Finally, we observed decreased movement of LC3B-GFP-containing structures in AP-4 ε KO neurons (Fig 8D and 8E and S1 Video), suggesting that delivery of ATG9A to pre-autophagosomal structures forming in the distal axon [31] contributes to their maturation and retrograde motility.

## Discussion

From a mechanistic standpoint, AP-4-deficiency syndrome belongs to a group of genetic diseases referred to as “coatopathies” because they arise from defects in protein coats involved in intracellular protein sorting [14]. These diseases are multisystemic, but often impact on the CNS, due to the greater vulnerability of neurons to alterations in protein trafficking [14]. Key to the understanding of the pathogenesis of these diseases is the identification of cargos that are missorted when the corresponding coat proteins are absent or inactive. In this study, we present evidence that the transmembrane autophagy protein ATG9A fails to exit the TGN towards the peripheral cytoplasm in skin fibroblasts from AP-4 μ4 deficient patients and neurons from AP-4 ε KO mice. This defect in ATG9A trafficking likely contributes to the motor and behavioral deficits observed in humans and mice with mutations in AP-4 subunit genes.

### AP-4 ε KO mouse is a suitable animal model for AP-4-deficiency syndrome

A previous study showed that AP-4 β4 KO mice performed poorly in the rotarod test, but did not report any other motor or behavioral abnormalities [18]. Here we show that AP-4 ε KO mice exhibit additional neurological defects, including hindlimb clasping, reduced grip strength, increased ambulation and enhanced acoustic startle response. Some of the neurological phenotypes of the AP-4 ε KO mice are consistent with the motor deficits observed in humans with AP-4-deficiency syndrome [6, 7, 8, 9, 10, 11, 12]. It is currently unclear, however, if the increased ambulation and startle response observed in the AP-4 ε KO mice have any correlates in AP-4-deficient patients. We were unable to detect defects in learning and memory in the AP-4 ε KO mice, in contrast to the intellectual disability that is characteristic of AP-4 deficiency in humans. This difference could indicate that the mutant mice lack cognitive defects, or that basic learning and memory tests in mice are not sensitive or specific enough for the type of intellectual disability present in the patients. Thus, AP-4 ε KO mice appear as a suitable animal model to investigate the pathogenesis of motor deficits in AP-4-deficient patients and to test the effectiveness of potential therapeutic approaches.

### Corpus callosum and neuroaxonal dystrophy in AP-4 ε KO mice

MRI and histopathological analyses revealed another characteristic of AP-4 ε KO mice that matches a feature of AP-4-deficient patients: the presence of a thin corpus callosum. The corpus callosum is a large tract of myelinated axons that connect the two cerebral hemispheres, integrating motor, sensory and cognitive functions [32]. A thin corpus callosum is also found in other forms of HSP, including SPG1, SPG7, SPG11, SPG18, SPG21, SPG32, SPG45, SPG46, SPG47, SPG48, SPG49, SPG52, SPG54, SPG56, SPG63, SPG65 and SPG71 [1, 3]. The proteins that are defective in all of these diseases, including AP-4, are likely required for the growth, guidance or maintenance of axons that, together with glial cells, make up the corpus callosum.

In addition to a thin corpus callosum, AP-4 ε KO mice exhibit axonal spheroids or swellings similar to those previously described in AP-4 β4 KO mice [18]. The presence of axonal spheroids in AP-4 ε KO mice is widespread throughout the CNS, including the cerebellum, hippocampus, midbrain and spinal cord. We have not further characterized the composition of these spheroids, other than by the variable presence of LAMP1, a protein that normally localizes to late endosomes, lysosomes and autolysosomes. In addition, DCN spheroids contain an accumulation of the glutamate receptor proteins δ2R and GluA2. However, the axonal spheroids in AP-4 ε KO mice appear similar to those observed in other human or mouse models of HSP, including SPG2 [33], SPG4 [34], SPG7 [35], SPG10 [36], SPG11 [37], SPG35 [38] and SPG79 [39]. AP-4 deficiency thus shares with a subset of HSPs the phenotypes of thin corpus callosum and widespread neuroaxonal dystrophy, suggesting that the corresponding proteins are all required for proper axonal development and function.

### ATG9A mislocalization and autophagic defects in AP-4 deficient mice and humans

Although δ2R and GluA2 were present in calbindin-positive DCN spheroids from AP-4 ε KO mice, GluA1 remained polarized to the somatodendritic domain of Purkinje neurons and did not accumulate in calbindin-positive DCN spheroids of AP-4 ε KO mice. This normal distribution of GluA1 was observed using two different antibodies and two different protocols for processing of the cerebellar sections prior to staining. In addition, both endogenous and transgenic GluA1 exhibited somatodendritic polarity and were absent from axonal swellings in cultured hippocampal neurons from AP-4 ε KO mice. Taken together, these findings support the notion that some glutamate receptor proteins accumulate in axonal spheroids in AP-4 ε KO mice, as previously reported for AP-4 β4 KO mice [18]. However, they also point to differential effects of AP-4 deficiency on the trafficking of AMPAR receptors with different subunit compositions. Nevertheless, we cannot rule out that differences in the distribution of GluA1 and GluA2 in AP-4 ε KO mice are due to recognition of different populations of AMPAR by the antibodies used in our study.

We next considered the possibility that the accumulation of glutamate receptors in axonal spheroids could result from more direct effects of AP-4 deficiency on autophagy. We focused our attention on ATG9A, a protein that was recently shown to behave as an AP-4 cargo in non-neuronal cells [19]. Indeed, we found that endogenous ATG9A is depleted from the peripheral cytoplasm and highly concentrated at the TGN in skin fibroblasts from patients with AP-4 μ4 mutations, the first instance in which an endogenous AP-4 cargo is shown to be missorted in patients’ cells. A similar alteration was observed in neurons from the hippocampus, cerebral cortex, cerebellum and spinal cord of AP-4 ε KO mice. The concentration of ATG9A at the TGN was made all the more evident by the elevated levels of ATG9A in the patients’ fibroblasts and various parts of the brain of KO mice. Upregulation of ATG9A could be part of a physiologic mechanism to compensate for the mislocalization of ATG9A in the mutant mice, ensuring that enough ATG9A escapes retention at the TGN to alleviate the autophagy defects.

ATG9A functions to deliver lipids or membranes to forming autophagosomes throughout the cytoplasm [40]. However, we did not detect obvious changes in the levels of endogenous LC3B-I/II and other autophagy proteins in the brain of AP-4 ε KO *vs*. WT mice. This was in contrast to non-neuronal cells, in which the conversion of LC3B-I to LC3B-II was impaired [19]. This cell-type specific behavior is consistent with previous studies showing differences in the regulation and functions of autophagy in neurons as compared to other cells [26, 27]. An important function of autophagy in neurons is the degradation of abnormal proteins and organelles [41, 42]. In line with this function, we found increased accumulation of huntingtin mutant aggregates in the axon of AP-4 ε KO hippocampal neurons. Therefore, AP-4-deficient neurons have an impaired ability to clear aggregated proteins from the axon, a common cause of neuronal degeneration [43]. This phenotype was reversed by overexpression of ATG9A-mCherry, consistent with the notion that impaired aggregate clearance is due to insufficient amounts of ATG9A-mCherry reaching sites of autophagosome formation. Moreover, we observed that LC3B-GFP structures in the distal axon were less mobile in AP-4 ε KO relative to WT hippocampal neurons. Since motility is related to maturation of autophagosomes [31], AP-4-dependent delivery of ATG9A to the axon is likely essential for maturation of axonal autophagosomes. Defects in autophagosome maturation and aggregate clearance might thus contribute to the neuroaxonal dystrophy observed in AP-4 ε KO mice and to the neurological symptoms of AP-4 deficiency in humans.

### Phenotypic overlap with other autophagy disorders

The phenotype of AP-4 deficient mice and humans overlaps with that of other conditions caused by mutations in components of the autophagy machinery. Particularly relevant is the finding that mice with a brain-specific KO of ATG9A exhibit poor roratod performance, dysgenesis of the corpus callosum, and calbindin-positive spheroids in DCN [44], similarly to the AP-4 ε KO mice described here. These observations support the functional connection of AP-4 with ATG9A. Nevertheless, ATG9A KO is more deleterious than AP-4 ε or β4 KO, as ATG9A KO die embryonically or perinatally, depending on the mouse strain and whether the KO is general or CNS-specific [44, 45, 46]. Complete absence of ATG9A thus has a worse outcome than its retention at the TGN. This could be because the compensatory increase in ATG9A levels in AP-4 ε KO neurons allows for some ATG9A to reach pre-autophagosomal structures and thus maintain a certain level of autophagy in the absence of AP-4.

Mutations in other autophagy proteins also cause some of the defects observed in AP-4-deficient humans and mice. For example, SPG49 is another form of complicated spastic paraplegia caused by mutation of the *TECPR2* gene encoding an LC3B-interacting protein [47]. SPG49 patients also exhibit thin corpus callosum and neuroaxonal dystrophy [47]. Likewise, patients with Vici syndrome, a multisystem disorder caused by mutations in the autophagosome-lysosome tethering protein EPG5 [48, 49, 50], have callosal agenesis, developmental delay, microcephaly, and seizures [51]. EPG5 KO mice also show degeneration of corticospinal tracts [52]. Finally, KO of genes encoding the autophagy proteins ATG5 [41], ATG7 [42], RB1CC1 29] or WDR45 [53] in the mouse all cause motor and behavioral abnormalities, axonal swellings, accumulation of neuronal inclusion bodies and axonal degeneration. These observations highlight common features between AP-4 deficiency and primary autophagy disorders, suggesting related mechanisms of pathogenesis and supporting another alternative classification of AP-4 deficiency syndrome as a congenital disorder of autophagy [54].

### Concluding remarks

Although all the available evidence points to a critical role of the connection of AP-4 with ATG9A and autophagy in the pathogenesis of AP-4 deficiency syndrome, additional scenarios should be considered. First, ATG9A has been proposed to have functions that are unrelated to autophagy [44, 55], although the mechanisms involved are unknown. Furthermore, AP-4 is likely responsible for the sorting of other cargos whose mislocalization might also contribute to neurological dysfunction. These cargos include members of the amyloid precursor protein (APP) family, APP, APLP1 and APLP2, which, like ATG9A [19], bind to AP-4 μ4 through a YXXØE sorting signal [17]. APP family members are involved in neuronal development, particularly axon growth and guidance [56]. Significantly, mice with mutations in the gene encoding APP show a high incidence of corpus callosum agenesis [57]. Finally, since AP-4 mediates ATG9A export from the TGN in multiple cell types, glial cell defects could also contribute to neurological dysfunction in AP-4 deficiency syndrome. In this regard, the X-linked SPG2 is caused by hemizygous mutations in the *PLP1* gene encoding the myelin proteolipid protein 1 [58], which is specific to oligodendrocytes and contributes to myelin sheath formation and axon survival.

Although the heterozygous parents of AP-4-deficient children do not present any clinical symptoms, rare heterozygous variants of AP-4 ε have been associated with persistent developmental stuttering, a disorder of speech volition [59]. We think that this condition might result from low-penetrance, mild dysfunction of some of the neuronal circuits affected by homozygous AP-4 deficiency.

The finding of ATG9A missorting and autophagic defects in AP-4-deficient cells offers new opportunities for the development of cell-based drug screens. For example, it should now be possible to screen for drugs that mobilize ATG9A from the TGN or that correct autophagic defects in AP-4-deficient cells [60]. Candidate drugs that are identified in these screens could be tested for their effectiveness in treating the symptoms of AP-4 deficiency in the AP-4 ε KO mice described here.

## Materials and methods

### Ethics statement

All animal procedures were conducted under protocol #15–021 approved by the NICHD Animal Care and Use Committee, in adherence to the NIH Guide for the Care and Use of Laboratory Animals. Fibroblasts from human patients and controls were obtained from GMSM, according to Erasmus MC institutional review board requirements (METC-2012387).

### Mice

*C57BL/6J* Ap4e1^tm1b(KOMP)Wtsi^ mice (indicated as tmb1 in Fig 1A), produced as described in ref. [61], were obtained from the UC Davis KOMP repository (https://www.komp.org/). These mice carry a deletion of exon 3 of the *AP4E1* gene. The tm1b mice were bred to a site-specific Flp deleter strain from The Jackson Laboratory (B6.Cg-Tg(Pgk1-flpo)10Sykr/J, Stock 011065/FLPo-10) to remove the LacZ and neomycin reporter cassette (Fig 1A). WT mouse counterparts were obtained from The Jackson Laboratory (C57BL/6J, Stock 000664/Black 6). Homozygous AP-4 ε -/- mutant (KO), heterozygous AP-4 ε +/- mutant, and homozygous AP-4 ε +/+ (WT) mice were obtained by breeding heterozygous mice. Littermates were used in most experiments. Mice were housed in groups no larger than four mice per cage with food and water *ad libitum* and under a 12 h light-dark cycle (6:00 on, 18:00 off). Shortly after weaning, genomic DNA was isolated from ear snips using a mouse genotyping kit from KAPA Biosystems. PCR was performed using primers for regions flanking exon 3: 5’- GCCTCTGTTTAGTTTGCGATG-3’ and 5’- TGACTCCAAAAGGATGCACA-3’. These primers amplified a 932 bp fragment from the WT allele and a 268 bp fragment from the KO allele. DNA amplification was performed using an initial denaturation (95°C for 3 min) followed by 30 cycles of denaturation (95°C, 15 s), annealing (60°C, 15 s) and extension (72°C, 15 s). All PCR reactions used KAPA Fast genotyping mix. The size of the PCR products was determined on agarose gels.

### Antibodies

The following antibodies were used in this study: rabbit anti-ATG9A (Abcam, cat. ab108338, 1:200 for immunofluorescence (IF), 1:1,000 for immunoblotting (IB), rabbit anti-LC3B (Sigma-Aldrich, cat. L7543, 1:1,000 for IB), mouse anti-AP-4 ε (BD Biosciences, cat. 612018, 1:75 for IF, 1:400 for IB), rabbit anti-AP-4 β4 (C terminus) generated in our laboratory (anti-β4C, ref. [4], 1: 500 for IB), mouse anti-AP-1 γ1 (BD Biosciences, cat. 610385, 1:2,500 for IB), sheep anti-TGN46 (Bio-Rad, cat. AHP500G, 1:500 for IF), mouse anti-GM130 (BD Biosciences, cat. 610822, 1:250 for IF), chicken anti-hemagglutinin (HA) (Millipore, cat. ab3254, 1:250 for IF), mouse anti-α-tubulin (Sigma, cat. T9026, 1:1,000 for IB), rabbit anti-β-tubulin (Cell Signaling, cat. 2146, 1:2,500 for IB), mouse anti-calbindin (Abcam, clone CB-955, cat. ab82812, 1:1,000 for immunohistochemistry (IHC), rat anti-LAMP1 (Developmental Studies Hybridoma Bank, cat. 1D4B, 1:500 for IHC), rat anti-non-phosphorylated neurofilament H (BioLegend, clone SMI-32, cat. 801701, 1:500 for IHC), rabbit anti-glutamate receptor 1 (AMPA subtype GluA1) (Abcam, cat ab31232, 1:100 for IF, 1:100 for IHC, and Millipore, cat AB1504, 1:300 for IHC), rabbit anti-glutamate receptor 2 (AMPA subtype GluA2) (Sigma, SAB4501295, 1:100 for IF, 1:100 for IHC), rabbit polyclonal anti-GluD2-C (δ2R) (Frontier Institute Co., ltd, 897-934/ Rb-Af1200, AB_2571601, 1:300 for IHC), mouse HRP-conjugated anti-GAPDH (Santa Cruz, clone 0411, cat. sc-47724, 1:500 for IB), rabbit anti-GABARAP (Abcam, clone EPR4805, cat. ab109364, 1:1,000 for IB), rabbit anti-ATG5 (Cell Signaling, clone D5F5U, cat. 12994, 1:1,000 for IB), rabbit anti-ATG7 (Cell Signaling, clone D12B11, cat. 8558, 1:1,000 for IB), guinea pig anti-SQSTM1 (MBL, cat. PM066, 1:1,000 for IB), Alexa Fluor 488-conjugated donkey anti-rabbit IgG (Invitrogen, cat. A21206, 1:1,000), Alexa Fluor 488-conjugated donkey anti-mouse IgG (Invitrogen, cat. A21202, 1:1,000), Alexa Fluor 555-conjugated donkey anti-mouse IgG (Invitrogen, cat. A31570, 1:1,000), Alexa Fluor 405-conjugated donkey anti-mouse IgG (Invitrogen, cat. A31553, 1:1,000), Alexa Fluor 647-conjugated donkey anti-mouse IgG (Invitrogen, cat. A31571, 1:1,000), Alexa Fluor 555-conjugated donkey anti-sheep IgG (Invitrogen, cat. A21436, 1:1,000), Alexa Fluor 647-conjugated goat anti-chicken IgG (Invitrogen, cat. A21449, 1:1,000), HRP-conjugated donkey anti-rabbit IgG (GE Healthcare, cat. NA934V, 1:5,000), HRP-conjugated sheep anti-mouse IgG (GE Healthcare, cat. NXA931, 1:5,000), and HRP-conjugated donkey anti-guinea pig IgG (Jackson Immuno Research, cat. 706-035-148, 1:5,000).

### Immunoblotting

Tissues were homogenized in 10 mM HEPES pH 7.5, 150 mM NaCl, 1mM EDTA, 1% v/v Triton X-100 supplemented with protease inhibitors (Roche) using a Polytron PT2500E homogenizer (Fisher Scientific). Alternatively, cortical neurons in primary culture were incubated for 5 h in the presence or absence of 100 nM bafilomycin A1 (Sigma) added to the culture medium. Cells were then scraped from the plate in phosphate-buffer saline (PBS) pH 7.5, 0.5% v/v Triton X-100 supplemented with protease inhibitors, incubated on ice for 30 min, and centrifuged at 16,000 x g for 10 min. The supernatant was than transferred to a fresh tube. Human fibroblasts plated on 100-mm dishes were lysed in 0.8 ml of 50 mM Tris/HCl pH 7.4, 0.8% v/v Triton X-100, 75 mM NaCl supplemented with protease inhibitors (EDTA-free Complete; Roche). Protein concentration was measured using the Bio-Rad Bradford protein assay reagent. Samples were denatured at 95°C or 50°C (for ATG9A) for 5 min in Laemmli sample buffer (Bio-Rad) containing 2.5% v/v 2-mercaptoethanol (Sigma-Aldrich), then resolved by SDS-PAGE and transferred onto nitrocellulose or Immobilon-P (Millipore) membranes. Membranes were blocked using 5% bovine serum albumin (BSA) (Sigma Aldrich) or 3% dry milk (BioRad) in Tris-buffered saline (TBS) (KD Medical) containing 0.1% Tween 20 (Sigma-Aldrich), probed with different primary and HRP-conjugated secondary antibodies, and revealed with either SuperSignal West Dura Extended Duration Substrate (Thermo Fisher) or Western Lighting Plus (PerkinElmer, Inc.).

### Motor and behavioral tests

Young WT and AP-4 ε KO mice (PND 1–18) were initially screened using the SHIRPA protocol for general health measures [62] and found not to differ. Cohorts of 8–25 adult (2–8 month old) male and female mice were subsequently tested in a battery of behavioral assays to examine motor, motorsensory and higher order neurological functions, including clasping, grip strength, rotarod, open field, startle response, elevated plus maze, spontaneous T-maze and Barnes maze. Animals were sex and age matched for each experiment. D’Agostino normality test was used to calculate the number of mice needed per group and for subsequent statistical analysis using t-test and two-way ANOVA for repeated measures followed by Bonferroni post-hoc test.

Clasping. WT and KO mice were suspended by the tail for 15 s at 20 cm height from the procedure table and the posture of hindlimbs was visually examined. The response was considered clasping if one or both hindlimbs were retracted and touched the abdomen for more that 3 s during the suspension. On the other hand, if the hindlimbs remained splayed outward during the entire time of suspension, we considered the mouse as non-clasping.

Grip Strength. To evaluate neuromuscular function, we assessed the maximal grabbing force (in Newton, N) exerted by mice when pulled out of a 45° grid connected to a force meter (BIO-GS3; Bioseb). The grip strength of all four paws was tested at the same time in three consecutive measurements per mouse, and the grip strength calculated as the average of each measurement.

Rotarod. The accelerating rotarod test was used to assess balance and motor coordination [63]. Briefly, WT and KO mice were placed on a five-lane rotarod device (ENV-574M, Med Associates Inc.). Mice were started at 4 RPM for 10 s, and speed was progressively accelerated to 40 RPM in 5 min. An infrared beam at the bottom of the rotor automatically recorded the time when mice fell from the rod. Rotarod performance was scored in three consecutive trials per mouse.

Open Field. Novelty-induced locomotor activity of WT and AP-4 ε KO mice was recorded in an illuminated (200 lux) white square arena (50 x 50 cm, 35 cm high walls) every 5 min for a total of 30 min. Horizontal locomotor activity was video recorded and distance traveled scored using Any-Maze software V5.1 (Stoelting Co). The open field arena was wiped with 70% v/v ethanol between trials.

Startle response. Acoustic startle response was measured during a 30 min session in Plexiglas cylinders in ventilated sound-attenuating chambers and with 65 dB white noise background (San Diego Instruments). After a 5-min habituation period, WT and KO mice were presented with a total of 260 trials with pseudo-randomized inter-trial periods (5–25 s) consisting of acoustic startle trials with white noise bursts of various intensities (65 to 120 dB; 10 trials per intensity). In each trial, the highest startle intensity peak (in arbitrary units designated by the apparatus) was measured during the 100 ms interval after the startle stimulus, from which the individual mean highest startle intensity peak during the 100-ms null-period was recorded.

Elevated Plus Maze. The elevated plus maze was used to assess basal anxiety and risk-taking behaviors. Experiments were performed on a Plexiglas plus-shaped maze containing two dark enclosed arms and two open arms elevated 50 cm above ground. Each of the four arms was 30 x 5 cm and were connected to a 5 x 5 cm center arena, and the walls of the closed arms were 20 cm high. Trials started by placing WT and KO mice in the center of the maze and the exploratory activity was tracked by video recording for 5 min. The total time mice spent in the closed/open arms, and at the center of the maze, were scored by Any-Maze software (Stoelting). The plus maze was cleaned between trials with 70% v/v alcohol.

T-Maze. Short-term spatial memory was assessed in a T-maze to test for spontaneous exploratory working memory behaviors, as previously described [64]. Briefly, the T-shaped maze consisted of three 23 cm-long arms. The mouse was placed in the “start arm” and allowed to explore any of the remaining two “goal arms” of its preference. Once a mouse entered either the left or right goal arm (four paws and tail inside an arm), a door to restrict the mouse exploration to this arm was lowered during 30 sec. The mouse was immediately returned to the start arm and allowed again to freely explore the apparatus. If during the second trial the mouse decided to explore the previously unvisited remaining arm, “alternation” of behavior was recorded. Three sessions were recorded per mouse with a delay time of 90 min each, and percentage of alternation was scored.

Barnes Maze. The Barnes maze test was conducted as previously described [65]. The apparatus consisted of a white circular platform (92 cm diameter) with 20 equally spaced holes (5 cm diameter, 7.5 cm between holes) along the perimeter and elevated 100 cm above the floor. One hole was designated as escape hole, and provided access to an escape box containing a metal stairway for easy access that was not visible unless mice approached the hole closely. During the test, mice received aversive reinforcements (85 dB background noise and 900 lux bright light) to encourage escape behaviors from the maze. Following a habituation period (30 min), the test consisted of two phases: training (spatial acquisition) and testing (probe trial). The training phase consisted of 4 trials/day/mouse during four consecutive days with inter-trial periods of 30 min each. The duration of each trial was 3 min. During the training phase, both the time that the mice spent finding the target box (latency time) and the number of errors (nose pokes in any no target hole) for each trial were scored from day 1 to 4. The testing phase was run 24 h after conclusion of the training phase (day 5), and consisted of one single trial of 90 s of duration per mouse. In this case, the target box was taken away from the maze so that the mouse was unable to escape from the maze and the number of nose pokes in the escape/incorrect holes and the time in the correct zone (¼ of the platform area including two incorrect holes at each side of escape) were recorded. Latency time, number of nose pokes in holes and time in the correct zone were automatically measured with AnyMaze program (Stoelting Co.). The chamber was wiped between trials with a 70% v/v alcohol.

### Magnetic resonance imaging

MRI was performed as per ref. [66]. MRI data were acquired using a 14.1 T magnetic resonance microimaging system with an 89-mm vertical superconducting magnet (Bruker Spectrospin) and a Bruker Avance III NMR imaging console controlled by Paravision 5.1 software (Bruker Instruments) with a 35 mm ID actively shielded gradients. A 20 mm ID birdcage volume RF resonator was used in these experiments. 3D MR images were acquired using a standard multi-echo spin-echo 3D RARE sequence with an echo time (TE) of 6.837 ms and a repetition time (TR) of 200 ms, a RARE factor of 2, 1 average, and an acquisition bandwidth of 119.047 kHz. The image field of view was 20 x 13 x 13 mm with an imaging data matrix of 400 x 256 x 256. This produced a 50 μm isotropic image for the prescribed FOV. The k-space image data were processed by zero-filling to an 800 x 512 x 512 matrix before trapezoid k-space filtration, cutoff (0.25, 0.75), and sliding window k-space baseline correction before Fourier transformation to a final image set of 25 μm isotropic digital resolution.

### Histology

Paraffin embedding, sectioning, and H&E staining of WT and KO brains were performed by Histoserv, Inc. (Germantown, MD).

### Immunohistochemistry of brain sections

For immunostaining of tissue sections, mice were transcardially perfused via the left ventricle with 50 ml of phosphate-buffer saline (PBS) pH 7.5, followed by 40 ml of 4% v/v paraformaldehyde (PFA) in PBS. Brains were removed and post-fixed for an additional 16 h by immersion in 4% PFA at room temperature followed by storage in PBS. Subsequently, the brains were transferred to 30% sucrose in 0.1 M phosphate buffer (PB) overnight. Sagittal free-floating sections (35 μm thick) were prepared on a Leica 9000s microtome. For GluA1 (Millipore AB1504) staining, sections were additionally treated for 30 minutes at 85°C in 10 mM sodium-citrate buffer for antigen retrieval before blocking. For δ2R (Frontier Inst. 897–934, RB-Af1200) staining, sections were incubated in 1 mg/ml pepsin (DAKO) in 0.2 M NH_4_Cl for 10 min at 37°C for antigen retrieval. Sections were rinsed in 0.1 M PB and subsequently blocked with 4% normal goat serum in 0.1 M PB containing 0.25% v/v Triton X-100 (blocking solution) at room temperature for 2 h. Primary antibodies were diluted in blocking solution and the sections were incubated overnight at 4°C with gentle agitation. After washing 3 times with wash buffer (0.1 M PB containing 0.25% Triton X-100), sections were incubated for 2 h at room temperature with appropriate fluorophore-conjugated secondary antibodies (AlexaFluor 488 and 594) diluted in wash buffer. After an additional three washes with wash buffer, sections were mounted on coverslips with MOWIOL/DABCO containing 4',6-diamidino-2-phenylindole (DAPI). Confocal microscopy images were collected using an Olympus confocal microscope with a Plan Apochromat 63x objective (N.A. 1.40).

### Culture of human skin fibroblasts

Skin fibroblasts from a control individual (85E0344) and from two SPG50 patients carrying a donor splice site pathogenic mutation in intron 14 of the *AP4M1* gene (c.1137+1G—>T) (Patient 1: 87RD38 and Patient 2: 87RD39) [7] were cultured in Dulbecco’s modified Eagle’s medium (DMEM) (Gibco) containing 10% FBS (Corning), 2 mM L-glutamine (Gibco), 100 units/mL of penicillin, and 100 μg/ml streptomycin (Gibco) at 37°C in a 5% CO atmosphere. The two patients also carried heterozygous and homozygous mutations in the *ATS* gene, respectively, but these mutations did not account for the SPG50 phenotype [7]. Cells were used for immunoblotting and immunofluorescence microscopy at passage 10.

### Preparation, culture and transfection of neurons

Primary hippocampal neurons were prepared from 18.5-day-old (E18.5) WT and AP-4 ε KO mouse embryos as previously described [67]. Cortical neurons were prepared by an adaptation of the protocol for hippocampal neurons. In brief, after decapitation, the cortex from E18.5 mice was dissected and treated with 0.25% trypsin (Corning) and 100 μg/ml DNAse in 2.2 mM EDTA for 15 min. The tissue was then disrupted by pipetting up and down 10 times with 10 ml and 5 ml pipets. The cells were filtered through a 70 μm cell strainer and centrifuged for 5 min at 900 x g. The pellet was resuspended in 10 ml plating medium consisting of Dulbecco’s Modified Eagle Medium (DMEM) (with 4.5 g/l glucose, 25 mM HEPES, without phenol red) supplemented with 10% heat-inactivated horse serum, 100 U/ml penicillin and 100 μg/mL streptomycin. 80,000 cells per well were plated on 12-well plates previously coated with polylysine and laminin. After 4 h, the medium was changed to complete Neurobasal medium (CNB) consisting of Neurobasal medium (with phenol red) (Gibco), supplemented with B27 serum-free (Gibco), 100 U/ml penicillin and 100 μg/mL streptomycin.

Plasmids used for transfection encoded LAMP1-GFP [22], plasma membrane-red fluorescence protein (PM-RFP) (FYN-FKBP1A with a C-terminal RFP, gift from J. Lippincott-Schwartz), HTT103Q-GFP (Addgene #1385), Rab3A-GFP (cloned from Origene SC319905), ε-HA (pCI-neo-(HA)3-epsilon, ref. [19]), and ATG9-mCherry (ATG9 with a C-terminal mCherry, generated in our lab). Neurons plated on 12-well plates were transfected with 2–3 μg of DNA per well using 1.3 μL of Lipofectamine 2000 reagent (Invitrogen) in 200 mL of Opti-MEM I (Gibco) at DIV4. Approximately 1 h after transfection, the medium was replaced. Cells were cultured for 9–12 days before fixation.

### Immunofluorescence microscopy

Human skin fibroblasts and primary mouse hippocampal neurons were fixed for 18 min in 4% v/v PFA in PBS supplemented with 0.1 mM calcium chloride and 1 mM magnesium chloride (PBS-CM) containing 4% sucrose. Cells were then washed twice in PBS-CM and permeabilized with 0.2% v/v Triton X-100 for 15 min at room temperature, or 100% methanol for 5 min at -20°C for AP-4 ε staining. Primary and secondary antibodies were diluted in PBS-CM containing 0.2% gelatin and sequentially incubated for 30 min at 37°C. Coverslips were mounted with Fluoromount-G (Electron Microscopy Sciences). Confocal microscopy images were collected using a Zeiss LSM 780 confocal microscope with a Plan Apochromat 63x objective (N.A. 1.40).

### Live-cell imaging

For LysoTracker imaging, primary mouse hippocampal neurons, transfected at DIV4 with PM-RFP, were imaged at DIV8. The neurons were incubated for 40 min with LysoTracker Green DND-26 (Thermo Fisher) in CNB. Cells were washed twice with CNB before imaging. For DQ-BSA imaging, primary untransfected mouse hippocampal neurons at DIV8 were incubated with DQ Red BSA (Thermo Fisher) for 4 h. Neurons were then washed twice, incubated for 5 min with CellMask Deep Red Plasma membrane stain (Thermo Fisher), washed three more times, and then imaged live in an environmental chamber (temperature controlled at 37°C and CO_2_ at 5%) with an Eclipse Ti Microscope System (Nikon). NIS-Elements AR microscope imaging software was used for acquisition and Fiji (https://fiji.sc/) for processing. Spinning-disk confocal images were taken with a Plan Apo VC 60× objective (N.A. 1.40) and a high-speed electron-multiplying charge-coupled device camera (DU-897; Andor) mounted on the left portal. Movement of LC3B-positive vesicles was visualized in neurons transfected at DIV4 and imaged live at DIV8. Kymographs were generated with Fiji, using 5 min videos, from straightened lines (1 pixel thickness and 25 μm length) by re-slicing stacks followed by z-projection. The green channel was sequentially recorded every 1 second. The number of anterograde, retrograde or static events was determined manually from kymographs. Phase-contrast imaging of DIV8 hippocampal neurons in culture was performed on a Zeiss Axio Vert.A1 inverted microscope with a Plan Apochromat 40x objective (N.A. 0.55).

### Electron microscopy

For transmission electron microscopy, whole mice were anesthetized with isoflurane using a VetEquip Vaporizer until a toe pinch yielded no response, and transcardially perfused via the left ventricle with 50 ml of phosphate-buffer saline (PBS) pH 7.5, followed by 30 ml of 2% glutaraldehyde (GA), 2% formaldehyde (FA), 2 mM calcium chloride in PBS. Cerebellum samples of 1 mm^3^ were dissected, washed three times in 0.1M cacodylate pH 7.4 and further fixed in 2% GA, 2% FA, 2 mM calcium chloride, 0.1M cacodylate pH 7.4 for 2 h at 4°C, washed four times for 10 min in the buffer and postfixed in 2% osmium tetroxide in the same buffer for 2 h on ice. The samples were washed twice in the buffer, five times in water, stained *en bloc* overnight in 2% aqueous uranyl acetate and washed three times in water, dehydrated in a series of ethanol concentrations and penetrated with EMbed 812 (EMS, Hatfield, PA), which was polymerized for 60 h at 65°C in flat molds. Thin (80 nm) sections of the samples were cut on a Leica EM UC7 microtome (Leica, Deerfield, IL) and stained with uranyl acetate/lead citrate. The samples were examined on a FEI Tecnai 20 electron microscope operated at 120 kV, and images were recorded on an AMT XR81 CCD camera.

### Quantification and statistics

Mouse motor and behavioral data were analyzed using GraphPad Prism v6.0. All data were examined for normal distribution with D’Agostino-Pearson normality test. If data set exhibited normal distribution, unpaired two-tailed student t test or two-way ANOVA for repeated measures and Sidak’s multiple comparisons post-hoc analysis were used. Data were represented as the mean ± SEM., and significance was set at P<0.05. D/A polarity indexes were calculated as previously described [68]. Quantification of immunoblots, fluorescence microscopy, live-cell and H&E staining was represented as the mean ± SD or SEM from multiple determinations, as indicated in each figure. The significance for those experiments was calculated using unpaired two-tailed student t test and was set at P<0.05.

## Supporting information

S1 Fig AP-4 ε KO mice do not show deficits in learning, spatial/working memory and anxiety behaviors. (A) The elevated plus maze test was used to assess differences in basal anxiety and risk-taking behaviors. This test is based on the natural fear mice show to opened and elevated environments. Experiments are performed on a Plexiglas plus-shaped maze containing two dark enclosed arms and two open arms elevated 50 cm above ground. Hence, mice normally will avoid exploring the open arms of the maze, and differences in the time spent in the open/closed arms is considered as an index of their basal anxiety/risk taking behaviors. We did not observe behavioral differences in the time spent in the open and closed arms between KO (4 male, 4 female) and WT (5 male, 4 female) mice (P>0.05). (B) Barnes maze test. AP-4 ε KO mice (5 males, 6 females) and their control (WT) littermates (6 males, 6 females) were trained to escape from an environmentally aversive environment (92 cm diameter circular table elevated 100 cm above the floor, 900 lux bright white light, 85 dB white background) during four consecutive days using the Barnes maze to test for learning and spatial memory retrieval as previously described [65]. During the training sessions (days 1 to 4, left panels), KO and WT learned to a similar extent how to escape from the aversive environment by finding the exit hole fixed to intra- and extra-maze cues. Therefore, the latency time (time required to find the exit hole) and the number of incorrect attempts (errors) to escape from the aversive environment was progressively and similarly reduced for both KO and WT mice throughout the training days (P>0.05). Moreover, 24 h later after the last training session (day 5, right panel), mice were tested for memory retrieval by eliminating the exit hole. The seconds (s) spent around the exit area (correct zone) and number of incorrect attempts (errors) around the maze to find the exit were scored. Again, KO and WT mice did not show differences to remember the position of the exit hole (KO: 25.62 ± 2.07 s; WT: 20.53 ± 2.34 s; P>0.05) and had a similar number of errors (KO: 10.27 ± 1.74, WT: 14.92 ± 3.18, P>0.05). (C) T-maze test. Working memory was tested in KO (4 males, 4 females) and WT (5 males, 4 females) mice using the spontaneous alternation T-test as previously described [64]. This test is based on the natural tendency mice exhibit to explore novel environments. Briefly, if mice decide to explore one goal arm of the maze (left or right arm, starting from start arm) in the first trial, they naturally will choose the opposite non-visited goal arm in a consecutive trial. A successful alternation is scored if mice explore the two opposite goal arms in two consecutive trials. KO mice did not exhibit altered spontaneous alternation (working memory) as compared to WT mice (KO: 87.80 ± 6.09%, WT: 77.80 ± 5.55%, P>0.05). This figure is related to Fig 1G–1I.
(TIF)

S2 Fig The thickness of the cerebral cortex is unchanged in AP-4 ε KO relative to WT mice. (A) H&E staining of comparable coronal sections of brains from 9-month-old mice show similar thickness of the cortical layer of the cerebrum in WT and AP-4 ε KO animals. Results are representative of two mice per group. Bars: 500 μm. (B) Quantification of cortex thickness in three different cortical segments from comparable coronal brain sections of two WT and two AP-4 ε KO brains. Values are the mean ± SD. Differences are not significant. This figure is related to Fig 2B.
(TIF)

S3 Fig Unaltered number of Purkinje neurons in the cerebellum of AP-4 ε KO relative to WT mice. (A) Immunostaining of sections from the cerebellum of 8-month old WT and AP-4 ε KO mice using an antibody to endogenous calbindin. Images are shown in inverted grayscale. Bars: 100 μm. Images on the right column are 4-fold magnified views. Arrowheads indicate the Purkinje cell (Pc) layer. (B) Quantification of the number of Purkinje cells per 250 μm of linear cerebellar cortex in WT and AP-4 ε KO mice. Values are the mean ± SD from twelve Pc layer regions from each of three WT and two KO animals. No significant differences in the number of Pc in the cerebellum of KO vs. WT mice were observed. This figure is related to Fig 2E.
(TIF)

S4 Fig Axonal swellings visualized by immunohistochemistry, phase contrast and transmission electron microscopy. (A) Immunohistochemical staining shows the presence of spheroids containing LAMP1 (green) in sections from the hippocampus and spinal cord of AP-4 ε KO mice. Nuclei were stained with DAPI (blue). Bars: 50 μm (zoom-out view) and 10 μm (magnification). (B) Hippocampal neurons in primary culture were visualized at DIV8 by phase-contrast microscopy. Arrowheads show axonal swellings. (C) Quantification of the number of swellings per 100 μm of axon from neurons as in B. Values are the mean ± SD from 21 neurons for each genotype, ***P<0.0005. (D) Transmission electron microscopy showing myelinated axons from Purkinje neurons in deep cerebellar nuclei from WT and AP-4 ε KO mice. The bottom panels are magnifications of the boxed areas in the upper panels. Arrowheads indicate the myelin layer surrounding the Purkinje cell axons (green), and organelles with the appearance of lysosomes (red), mitochondria (orange) and tightly packed membrane cisternae (blue) inside the swellings. This figure is related to Fig 2C–2H.
(TIF)

S5 Fig Somatodendritic polarity of endogenous GluA1 in Purkinje neurons of the cerebellum from WT and AP-4 ε KO mice, demonstrated using different antibodies. Sections of the cerebellum from WT and AP-4 ε KO mice were co-immunostained with the antibodies ab31232 (Abcam) (A,B) or AB1504 (Millipore) (C,D) to the GluA1 subunit of AMPAR (green) together with an antibody to calbindin (red). Mo: molecular layer; Gr: granular layer, Pc: Purkinje cell layer, DCN: deep cerebellar nucleus. Nuclei were stained with DAPI (blue). Bars: 100 μm (A,C), 50 μm (B,D). Examples of spheroids are indicated by arrowheads. Single-channel images are shown in inverted grayscale. Notice the presence of GluA1 staining in the soma and dendritic field (Mo) and the absence of GluA1 staining in the axonal field (Gr) and DCN spheroids of calbindin-positive Purkinje neurons from both WT and KO mice. This figure is related to Fig 3C.
(TIF)

S6 Fig Somatodendritic polarity of endogenous GluA1 and transgenic GluA1-GFP in cultured hippocampal neurons from WT and AP-4 ε KO mice. (A) Immunostaining for the endogenous GluA1 subunit of AMPAR (ab31232 from Abcam) in hippocampal neurons from WT and KO mice. The entire neuronal body was highlighted by transfection with a plasmid encoding the plasma membrane marker PM-RFP. Arrowheads show the trajectory of the axon. Bars: 20 μm. (B) Segments of 50 μm from axons and dendrites from neurons in A were enlarged and straightened. (C) Hippocampal neurons from WT and KO mice were transfected with plasmids encoding GluA1-GFP (green) and CFP (cyan) and stained for the axon initial segment (AIS) with an antibody to ankyrin G (AnkG). The trajectory of the axon is indicated by arrowheads and a dashed box. Bars: 20 μm. In A-C, single-channel images are shown in inverted grayscale. (D) Quantification of dendrite/axon (D/A) polarity indexes from neurons such as those in A-C. Values are the mean ± SD from 10 neurons. D /A polarity indexes for endogenous GluA1 were 7.8 ± 2.9 in WT neurons and 7.6 ± 3.9 in AP-4 ε KO neurons (P>0.05). Polarity indexes for transgenic GluA1-GFP were 7.0 ± 2.9 in WT neurons and 7.4 ± 2.1 in AP-4 ε KO neurons (P>0.05). Notice that the somatodendritic polarity of endogenous GluA1 and and transgenic GluA1-GFP, is not changed in KO in comparison to WT neurons. This figure is related to Fig 3C.
(TIF)

S7 Fig Accumulation of ATG9A at the TGN of AP-4 μ4 mutant patient fibroblasts. (A) Skin fibroblasts from one control individual and two patients homozygous for mutations in the *AP4M1* gene encoding AP-4 μ4 [7] were immunostained for ATG9A, AP-4 ε, GM130 and/or TGN46, as indicated on the figure. Nuclei were stained with DAPI (blue). Bars: 20 μm. Notice the concentration of ATG9A at the TGN and its disappearance from the peripheral cytoplasm in the patient cells. These images are larger fields of cells shown in Fig 4B and 4C.
(TIF)

S8 Fig Distribution of endogenous ATG9A, transgenic LAMP1-GFP and transgenic RAB3A-GFP in axon and dendrites, and lysosome properties, in hippocampal neurons from WT and AP-4 ε KO mice. (A) Hippocampal neurons from WT and AP-4 ε KO mice were transfected with a plasmid encoding the plasma membrane marker PM-RFP alone or together with plasmids encoding the lysosomal marker LAMP1-GFP or the synaptic vesicle marker RAB3A-GFP. Endogenous ATG9A was detected by immunostaining. Notice the depletion of ATG9A from both axons and dendrites in neurons from AP-4 ε KO relative to WT mice. Also notice that the presence of LAMP1-GFP in both axon and dendrites and the axonal localization of RAB3A-GFP did not change in neurons from AP-4 ε KO relative to WT mice. (B,C) Hippocampal neurons from WT and AP-4 ε KO mice were stained with the acidic organelle marker LysoTracker (B) and lysosomal proteolysis marker DQ-BSA (C). Bars: 20 μm. Notice the similar staining of WT and AP-4 ε KO neurons by these lysosomal probes. This figure is related to Fig 5A and Fig 8A–8C.
(TIF)

S1 Video Decreased mobility of autophagosomes in the axon of AP-4 ε KO hippocampal neurons. Straightened 50 μm segments of the axon terminal from DIV8 WT and AP-4 ε KO hippocampal neurons expressing LC3B-GFP (top) were sequentially recorded every 1 second for 5 minutes. The corresponding kymographs are at the bottom. Note the retrogradely moving LC3B-GFP vesicles in the WT axon, and the absence of moving vesicles in the AP-4 ε KO axon. This video is related to Fig 8D.
(MOV)
