# Supplementary figures and images for: Altered distribution of ATG9A and accumulation of axonal aggregates in neurons from a mouse model of AP-4 deficiency syndrome
Source: PLoS Genet. 2018 Apr 26;14(4):e1007363. doi: 10.1371/journal.pgen.1007363 (PMC5940238; doi:10.1371/journal.pgen.1007363)

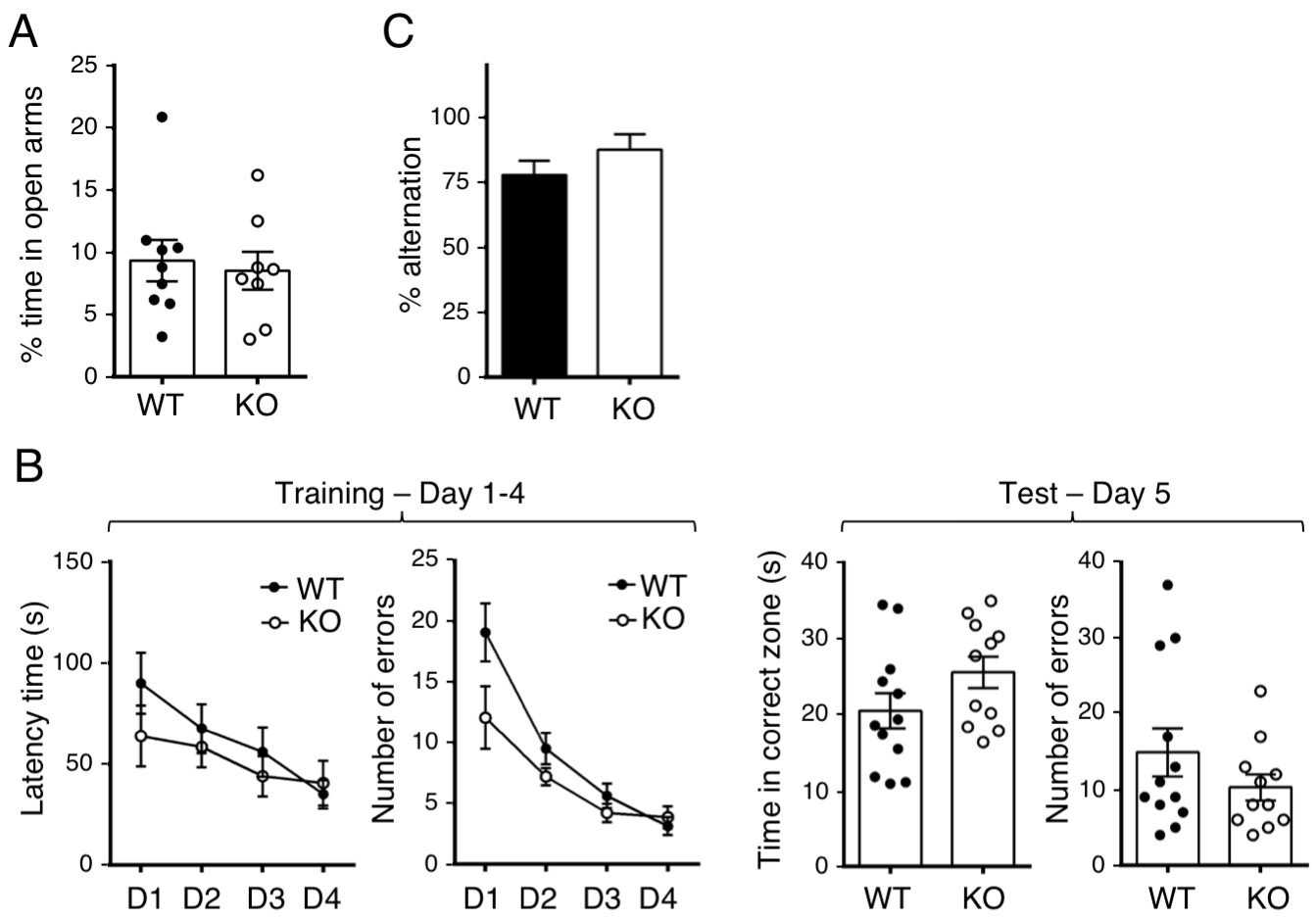

Supplement: S1 Fig — (A) The elevated plus maze test was used to assess differences in basal anxiety and risk-taking behaviors. This test is based on the natural fear mice show to opened and elevated environments. Experiments are performed on a Plexiglas plus-shaped maze containing two dark enclosed arms and two open arms elevated 50 cm above ground. Hence, mice normally will avoid exploring the open arms of the maze, and differences in the time spent in the open/closed arms is considered as an index of their basal anxiety/risk taking behaviors. We did not observe behavioral differences in the time spent in the open and closed arms between KO (4 male, 4 female) and WT (5 male, 4 female) mice (P>0.05). (B) Barnes maze test. AP-4 ε KO mice (5 males, 6 females) and their control (WT) littermates (6 males, 6 females) were trained to escape from an environmentally aversive environment (92 cm diameter circular table elevated 100 cm above the floor, 900 lux bright white light, 85 dB white background) during four consecutive days using the Barnes maze to test for learning and spatial memory retrieval as previously described [65]. During the training sessions (days 1 to 4, left panels), KO and WT learned to a similar extent how to escape from the aversive environment by finding the exit hole fixed to intra- and extra-maze cues. Therefore, the latency time (time required to find the exit hole) and the number of incorrect attempts (errors) to escape from the aversive environment was progressively and similarly reduced for both KO and WT mice throughout the training days (P>0.05). Moreover, 24 h later after the last training session (day 5, right panel), mice were tested for memory retrieval by eliminating the exit hole. The seconds (s) spent around the exit area (correct zone) and number of incorrect attempts (errors) around the maze to find the exit were scored. Again, KO and WT mice did not show differences to remember the position of the exit hole (KO: 25.62 ± 2.07 s; WT: 20.53 ± 2.34 s; P>0.05 [file pgen.1007363.s001.tif]

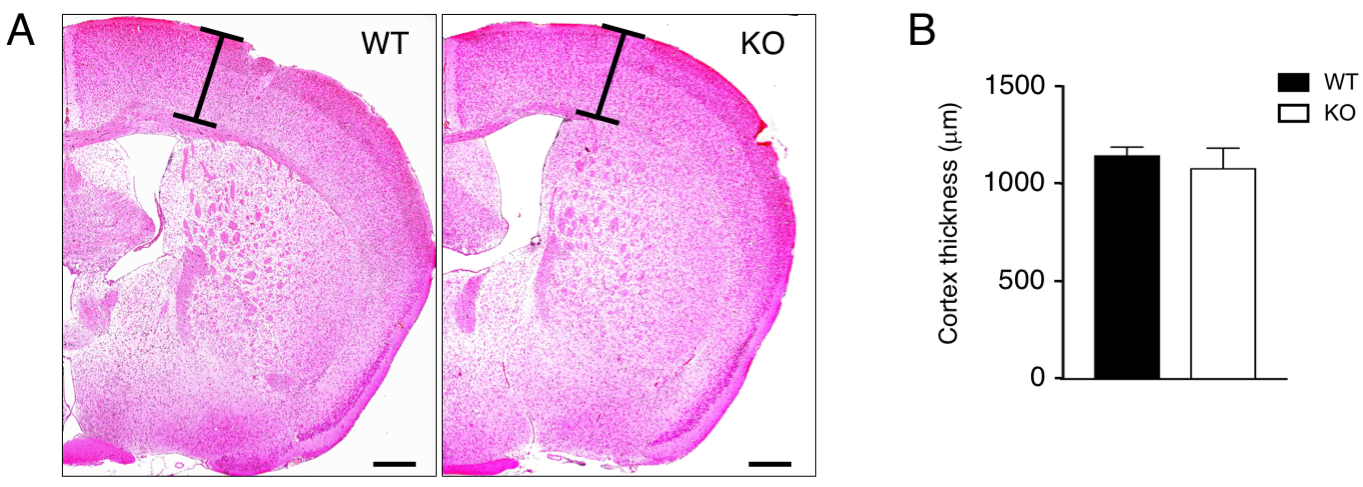

Supplement: S2 Fig — (A) H&E staining of comparable coronal sections of brains from 9-month-old mice show similar thickness of the cortical layer of the cerebrum in WT and AP-4 ε KO animals. Results are representative of two mice per group. Bars: 500 μm. (B) Quantification of cortex thickness in three different cortical segments from comparable coronal brain sections of two WT and two AP-4 ε KO brains. Values are the mean ± SD. Differences are not significant. This figure is related to Fig 2B. (TIF) [file pgen.1007363.s002.tif]

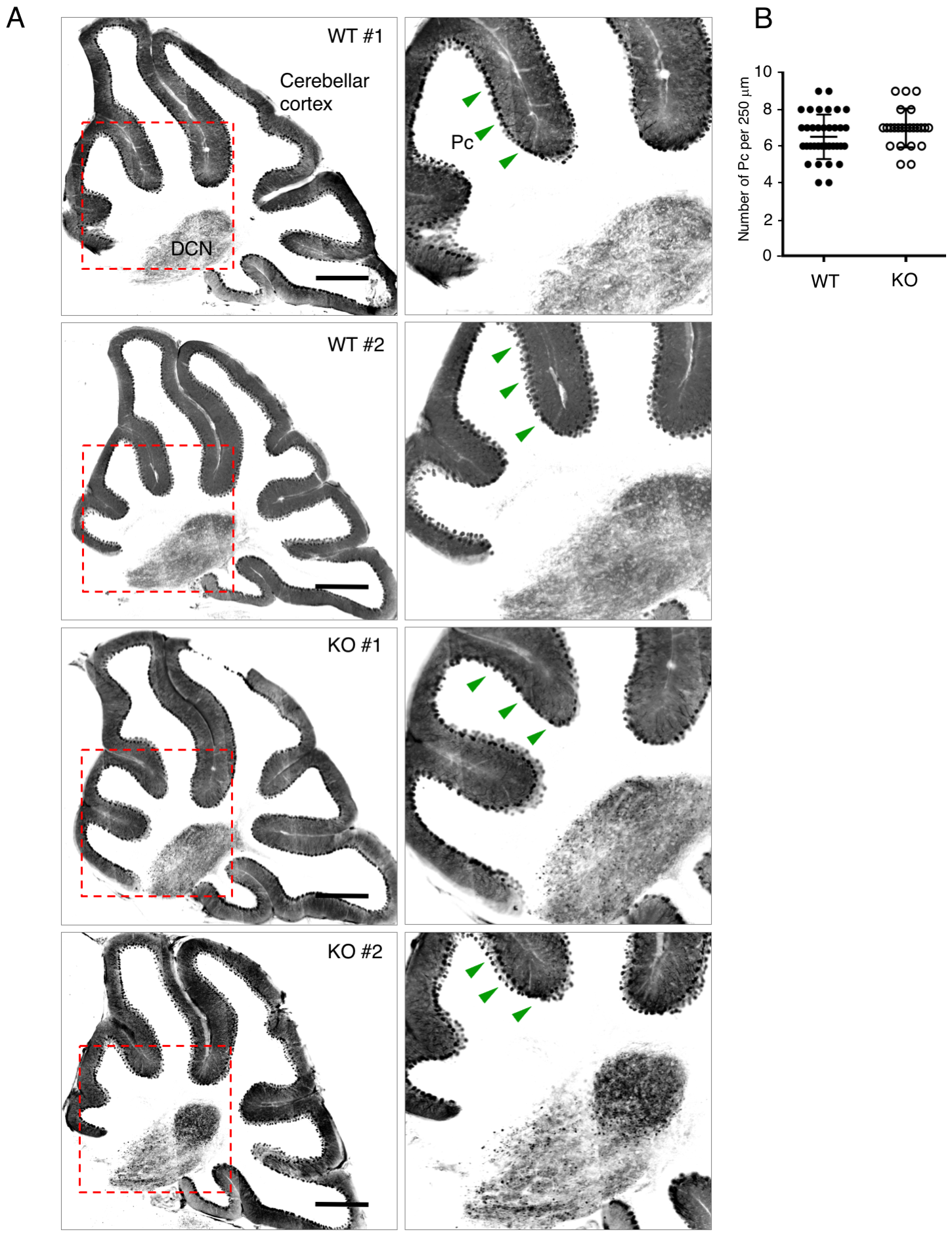

Supplement: S3 Fig — (A) Immunostaining of sections from the cerebellum of 8-month old WT and AP-4 ε KO mice using an antibody to endogenous calbindin. Images are shown in inverted grayscale. Bars: 100 μm. Images on the right column are 4-fold magnified views. Arrowheads indicate the Purkinje cell (Pc) layer. (B) Quantification of the number of Purkinje cells per 250 μm of linear cerebellar cortex in WT and AP-4 ε KO mice. Values are the mean ± SD from twelve Pc layer regions from each of three WT and two KO animals. No significant differences in the number of Pc in the cerebellum of KO vs. WT mice were observed. This figure is related to Fig 2E. (TIF) [file pgen.1007363.s003.tif]

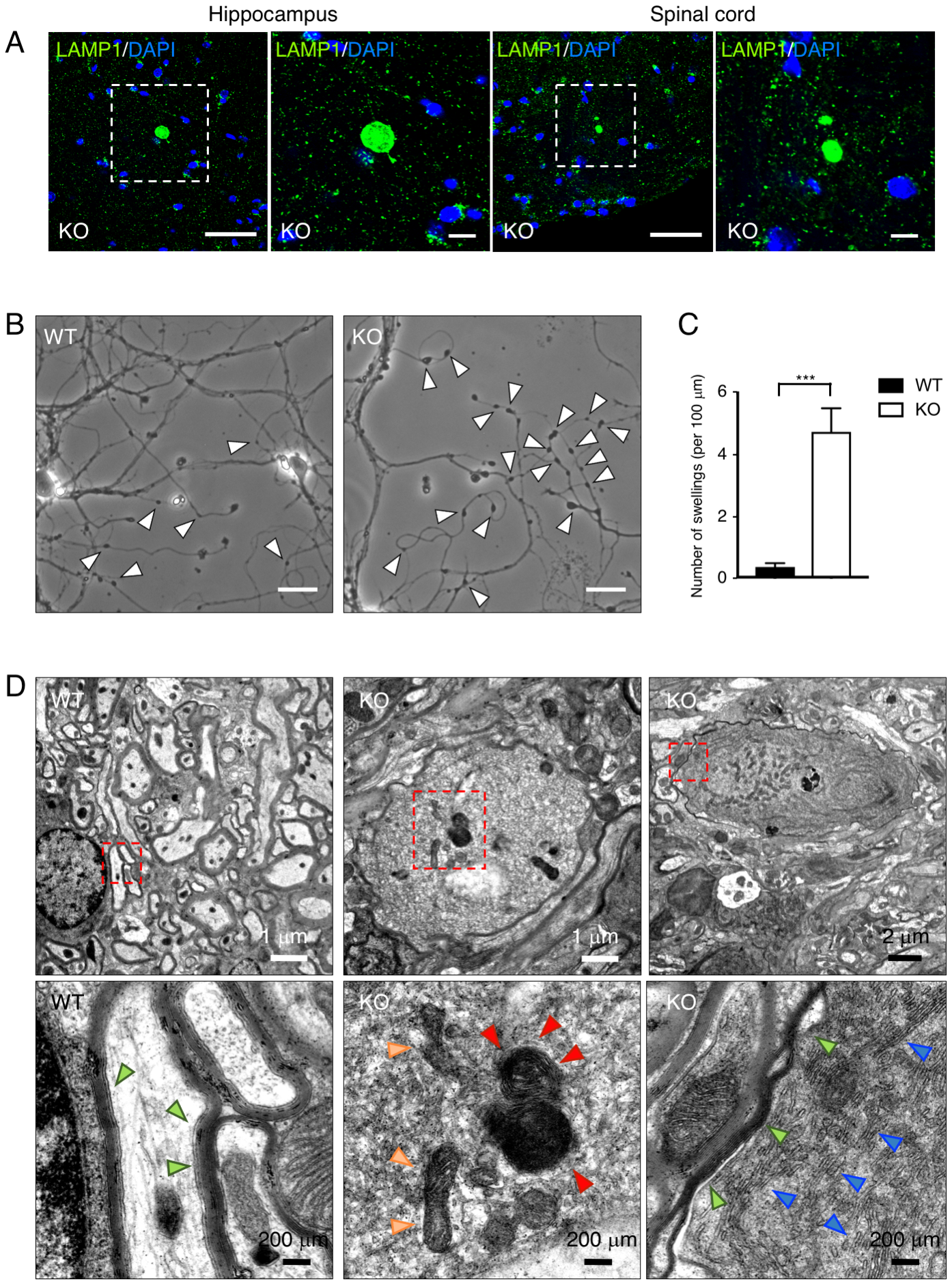

Supplement: S4 Fig — (A) Immunohistochemical staining shows the presence of spheroids containing LAMP1 (green) in sections from the hippocampus and spinal cord of AP-4 ε KO mice. Nuclei were stained with DAPI (blue). Bars: 50 μm (zoom-out view) and 10 μm (magnification). (B) Hippocampal neurons in primary culture were visualized at DIV8 by phase-contrast microscopy. Arrowheads show axonal swellings. (C) Quantification of the number of swellings per 100 μm of axon from neurons as in B. Values are the mean ± SD from 21 neurons for each genotype, ***P<0.0005. (D) Transmission electron microscopy showing myelinated axons from Purkinje neurons in deep cerebellar nuclei from WT and AP-4 ε KO mice. The bottom panels are magnifications of the boxed areas in the upper panels. Arrowheads indicate the myelin layer surrounding the Purkinje cell axons (green), and organelles with the appearance of lysosomes (red), mitochondria (orange) and tightly packed membrane cisternae (blue) inside the swellings. This figure is related to Fig 2C–2H. (TIF) [file pgen.1007363.s004.tif]

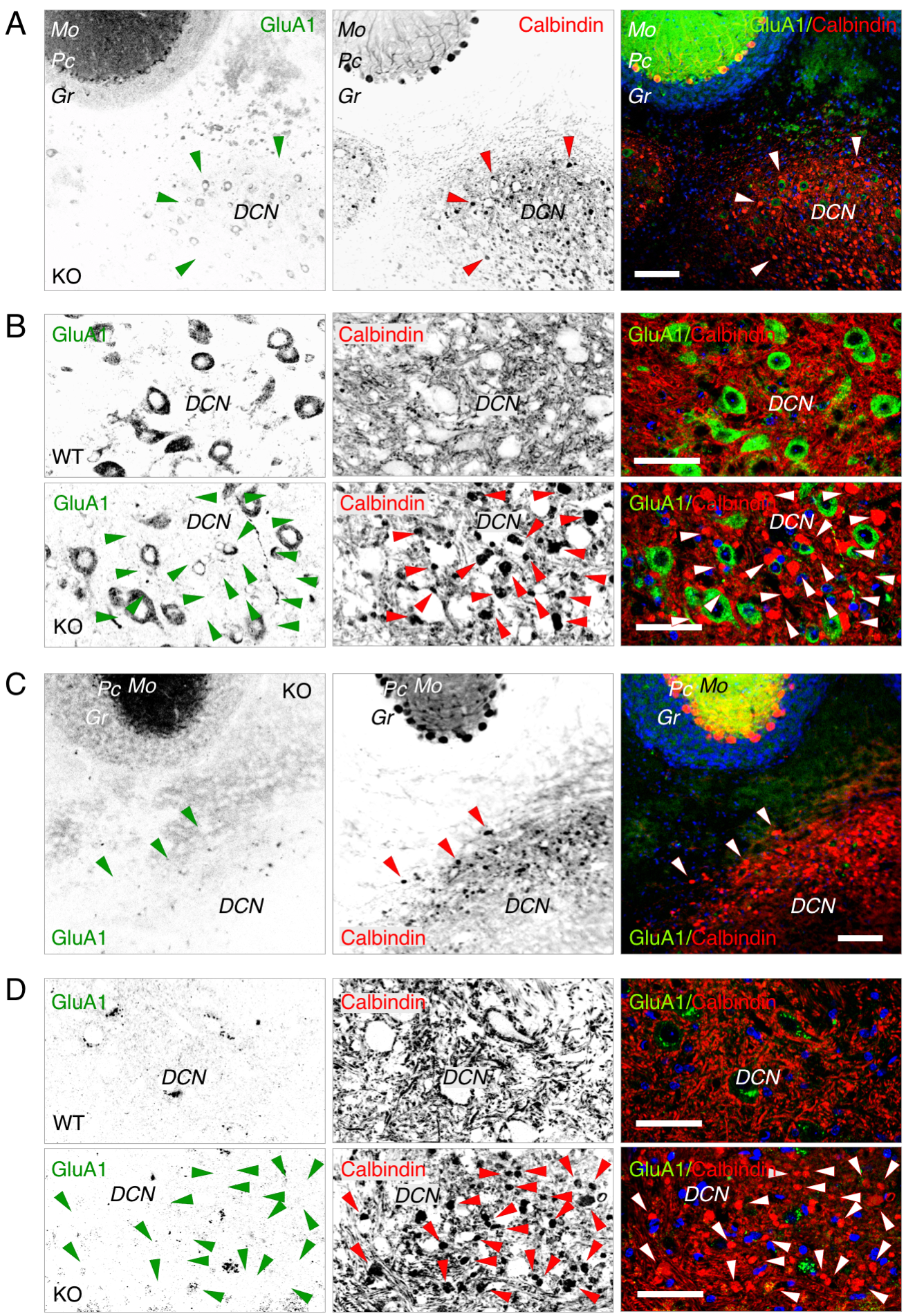

Supplement: S5 Fig — Sections of the cerebellum from WT and AP-4 ε KO mice were co-immunostained with the antibodies ab31232 (Abcam) (A,B) or AB1504 (Millipore) (C,D) to the GluA1 subunit of AMPAR (green) together with an antibody to calbindin (red). Mo: molecular layer; Gr: granular layer, Pc: Purkinje cell layer, DCN: deep cerebellar nucleus. Nuclei were stained with DAPI (blue). Bars: 100 μm (A,C), 50 μm (B,D). Examples of spheroids are indicated by arrowheads. Single-channel images are shown in inverted grayscale. Notice the presence of GluA1 staining in the soma and dendritic field (Mo) and the absence of GluA1 staining in the axonal field (Gr) and DCN spheroids of calbindin-positive Purkinje neurons from both WT and KO mice. This figure is related to Fig 3C. (TIF) [file pgen.1007363.s005.tif]

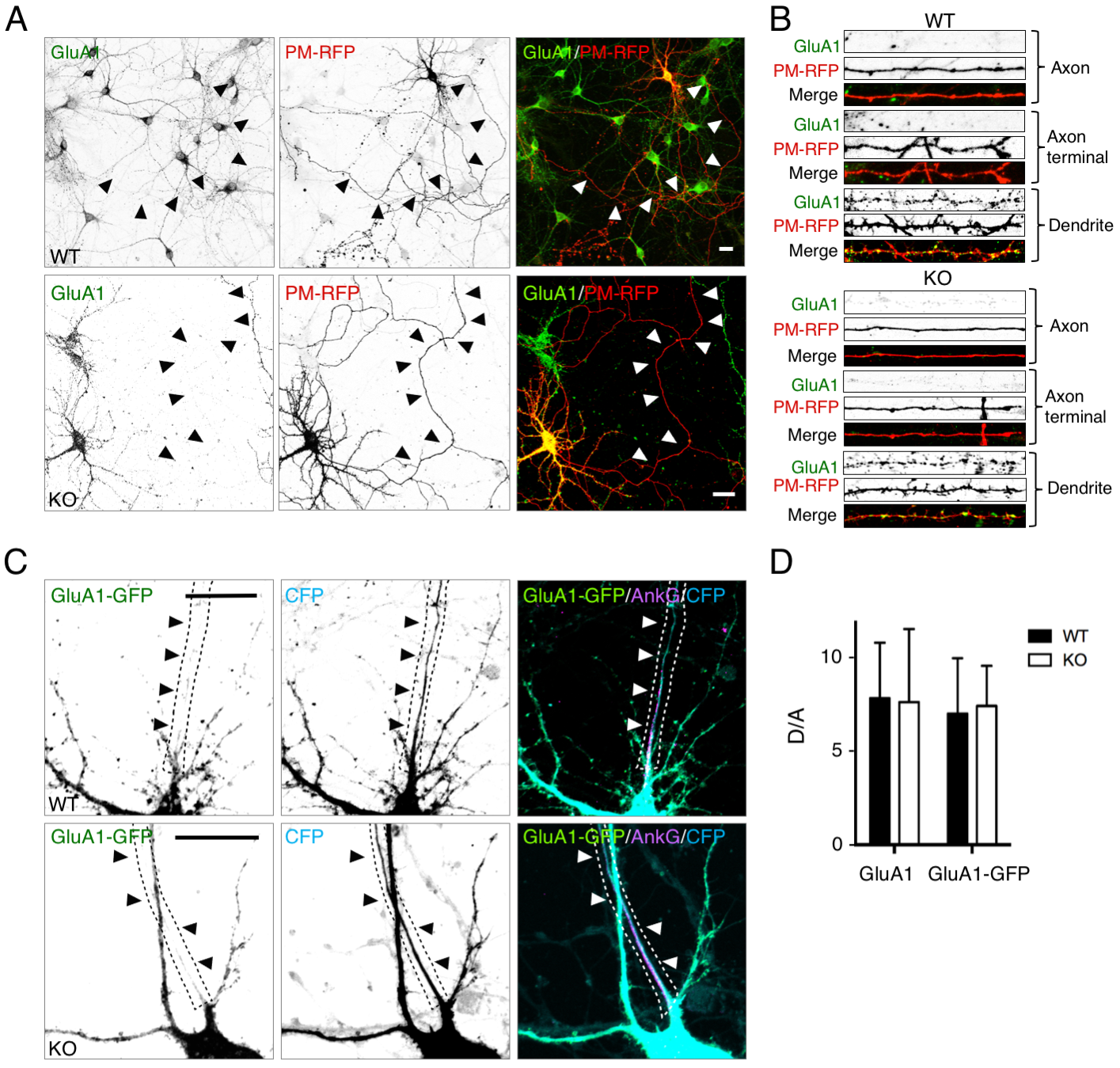

Supplement: S6 Fig — (A) Immunostaining for the endogenous GluA1 subunit of AMPAR (ab31232 from Abcam) in hippocampal neurons from WT and KO mice. The entire neuronal body was highlighted by transfection with a plasmid encoding the plasma membrane marker PM-RFP. Arrowheads show the trajectory of the axon. Bars: 20 μm. (B) Segments of 50 μm from axons and dendrites from neurons in A were enlarged and straightened. (C) Hippocampal neurons from WT and KO mice were transfected with plasmids encoding GluA1-GFP (green) and CFP (cyan) and stained for the axon initial segment (AIS) with an antibody to ankyrin G (AnkG). The trajectory of the axon is indicated by arrowheads and a dashed box. Bars: 20 μm. In A-C, single-channel images are shown in inverted grayscale. (D) Quantification of dendrite/axon (D/A) polarity indexes from neurons such as those in A-C. Values are the mean ± SD from 10 neurons. D /A polarity indexes for endogenous GluA1 were 7.8 ± 2.9 in WT neurons and 7.6 ± 3.9 in AP-4 ε KO neurons (P>0.05). Polarity indexes for transgenic GluA1-GFP were 7.0 ± 2.9 in WT neurons and 7.4 ± 2.1 in AP-4 ε KO neurons (P>0.05). Notice that the somatodendritic polarity of endogenous GluA1 and and transgenic GluA1-GFP, is not changed in KO in comparison to WT neurons. This figure is related to Fig 3C. (TIF) [file pgen.1007363.s006.tif]

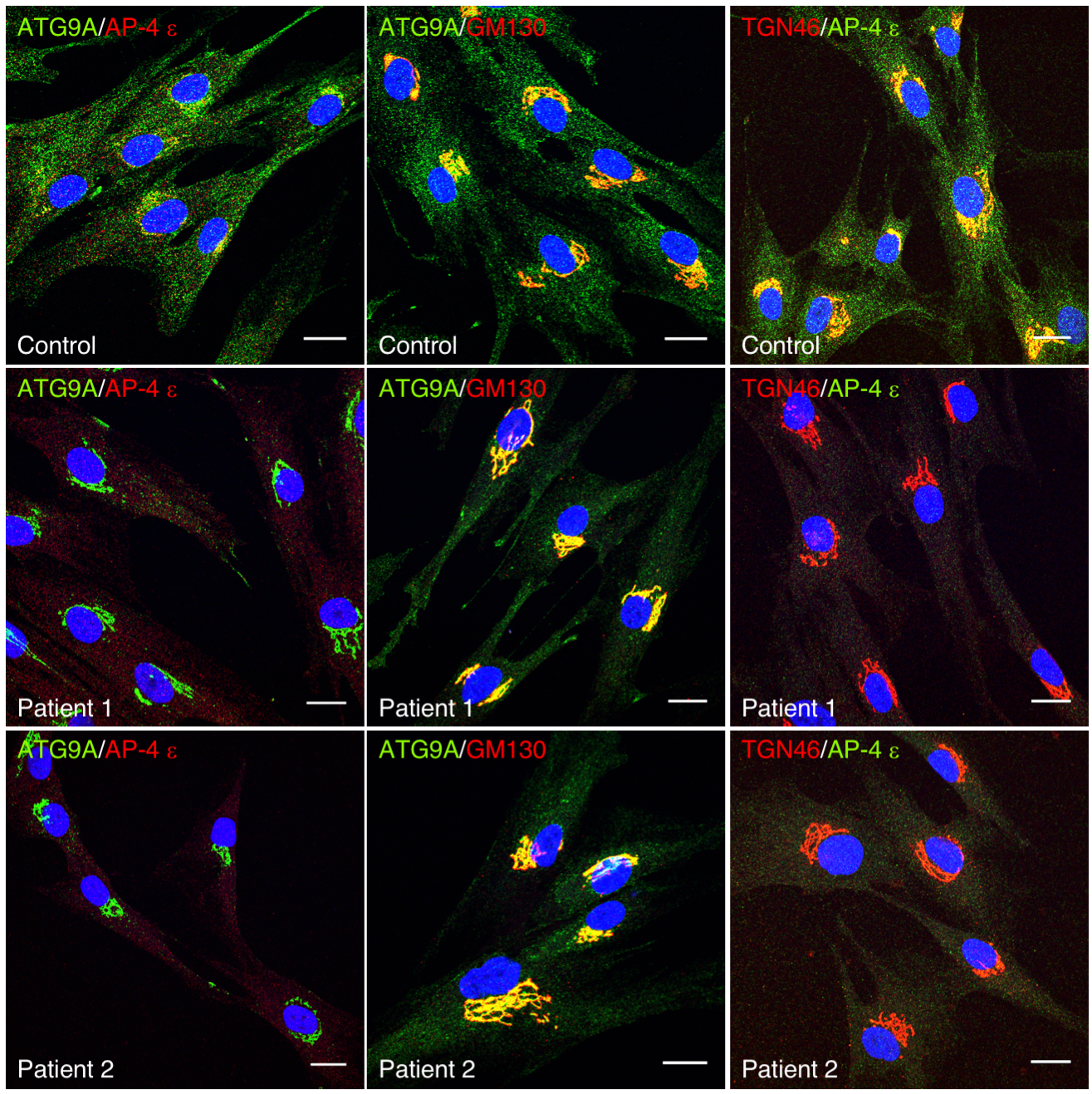

Supplement: S7 Fig — (A) Skin fibroblasts from one control individual and two patients homozygous for mutations in the AP4M1 gene encoding AP-4 μ4 [7] were immunostained for ATG9A, AP-4 ε, GM130 and/or TGN46, as indicated on the figure. Nuclei were stained with DAPI (blue). Bars: 20 μm. Notice the concentration of ATG9A at the TGN and its disappearance from the peripheral cytoplasm in the patient cells. These images are larger fields of cells shown in Fig 4B and 4C. (TIF) [file pgen.1007363.s007.tif]

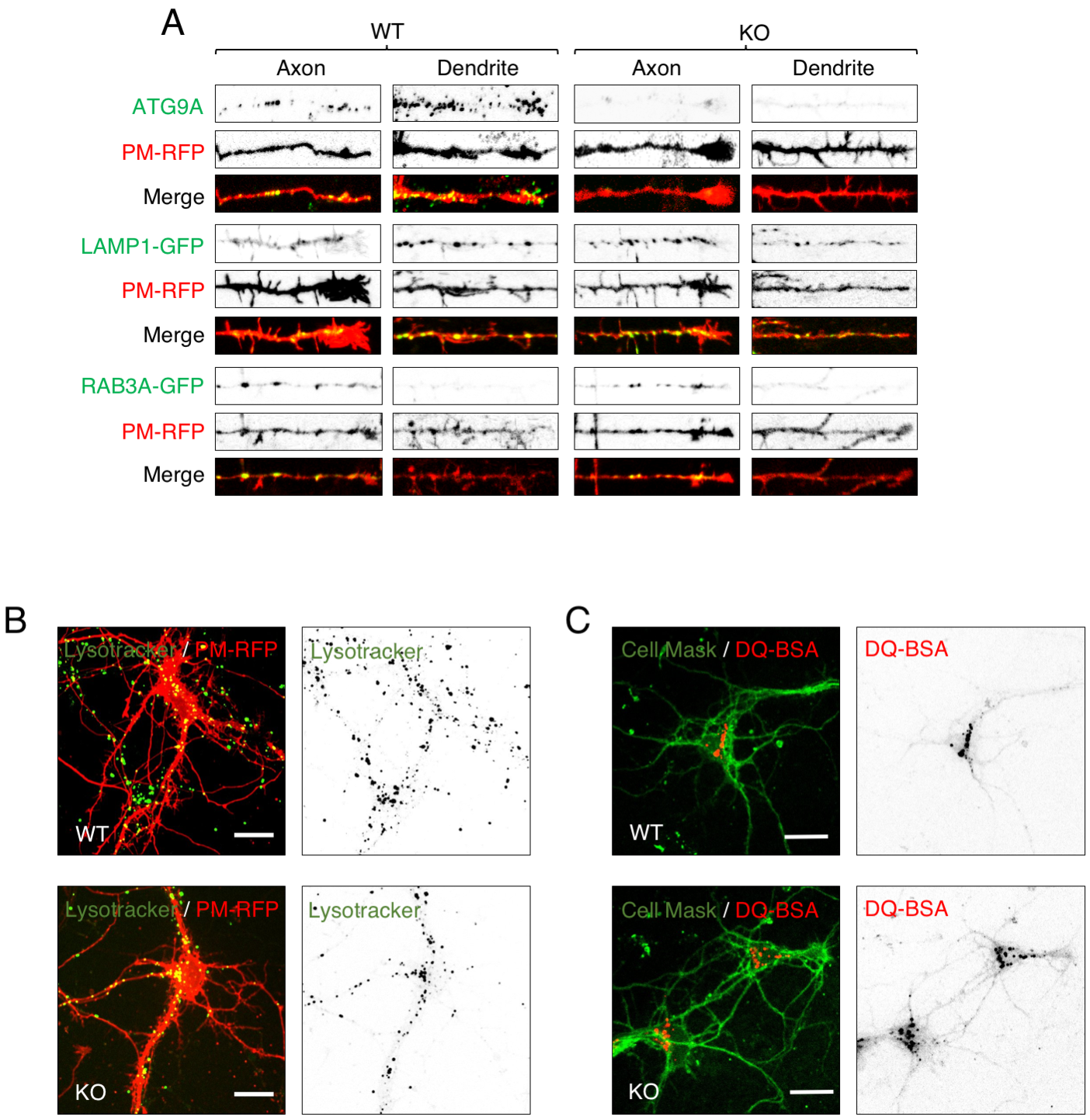

Supplement: S8 Fig — (A) Hippocampal neurons from WT and AP-4 ε KO mice were transfected with a plasmid encoding the plasma membrane marker PM-RFP alone or together with plasmids encoding the lysosomal marker LAMP1-GFP or the synaptic vesicle marker RAB3A-GFP. Endogenous ATG9A was detected by immunostaining. Notice the depletion of ATG9A from both axons and dendrites in neurons from AP-4 ε KO relative to WT mice. Also notice that the presence of LAMP1-GFP in both axon and dendrites and the axonal localization of RAB3A-GFP did not change in neurons from AP-4 ε KO relative to WT mice. (B,C) Hippocampal neurons from WT and AP-4 ε KO mice were stained with the acidic organelle marker LysoTracker (B) and lysosomal proteolysis marker DQ-BSA (C). Bars: 20 μm. Notice the similar staining of WT and AP-4 ε KO neurons by these lysosomal probes. This figure is related to Fig 5A and Fig 8A–8C. (TIF) [file pgen.1007363.s008.tif]
